# Hemodynamic factors of aortic dilatation after thoracic endovascular aortic repair for type-B aortic dissection

**DOI:** 10.3389/fbioe.2026.1780047

**Published:** 2026-04-22

**Authors:** Qian-hui Tang, Zhen Long, Ping Dai, Han Yang, Zhong Qin, Xuan-an Su, Yu-Lin Wang, Xing-ning Mao, Jing Chen, Hai-ying Zhang, Xiao Qin

**Affiliations:** 1 Department of Vascular and Endovascular Surgery, The First Affiliated Hospital of Guangxi Medical University, Nanning, Guangxi, China; 2 Department of Occupational Health and Environmental Health, School of Public Health, Guangxi Medical University, Nanning, Guangxi, China; 3 Department of Vascular and Endovascular Surgery, The First People's Hospital of Yulin, Yulin, Guangxi, China; 4 Department of Ultrasound, The First People's Hospital of Yulin, Yulin, Guangxi, China; 5 Center for Genomic and Personalized Medicine, Guangxi Key Laboratory for Genomic and Personalized Medicine, Guangxi Collaborative Innovation Center for Genomic and Personalized Medicine, Guangxi Medical University, Nanning, Guangxi, China; 6 Collaborative Innovation Centre of Regenerative Medicine and Medical BioResource Development and Application Co. constructed by the Province and Ministry, Guangxi Medical University, Nanning, Guangxi, China; 7 Guangxi Colleges and Universities Key Laboratory of Prevention and Control of Highly Prevalent Diseases, Guangxi Medical University, Nanning, Guangxi, China; 8 Department of Public Health, School of Medicine, Guangxi University of Science and Technology, Liuzhou, Guangxi, China

**Keywords:** aortic dilatation, computational fluid dynamics, hemodynamics, oscillatory shear index, thoracic endovascular aortic repair, type-B aortic dissection

## Abstract

**Objective:**

We aimed to investigate the hemodynamic factors associated with aortic dilatation following thoracic endovascular aortic repair (TEVAR) for type-B aortic dissection (TBAD).

**Methods:**

This retrospective study enrolled patients who developed aortic dilatation following TEVAR for TBAD, patients who did not develop aortic dilatation following TEVAR for TBAD, and healthy control subjects. Computed tomography angiography (CTA) images for the dilatation group were acquired at three time points: pre-TEVAR (Group A), 1-week post-TEVAR (Group B), and the most recent follow-up (Group C). For the non-dilatation group, CTA images were acquired pre-TEVAR (Group D) and 1 week post-TEVAR (Group E). The control group underwent a single CTA examination (Group F). Three-dimensional (3D) models were reconstructed from CTA images, and computational fluid dynamics (CFD) simulations were performed. The oscillatory shear index (OSI) at the ostia of the major arterial branches was defined as the primary CFD endpoint.

**Results:**

In the dilatation group, the pressures at the ostia of the brachiocephalic trunk, left common carotid artery, left subclavian artery, celiac trunk, superior mesenteric artery, bilateral renal arteries, inferior mesenteric artery, and bilateral common iliac arteries were lower in Group B than in Groups A and C. In the non-dilatation group, the pressures at the ostia of the brachiocephalic trunk, left common carotid artery, left subclavian artery, celiac trunk, superior mesenteric artery, left renal artery, inferior mesenteric artery, and right common iliac artery were higher in Group E than in Group D. The false lumen in Group B exhibited a larger region of high OSI compared with that in Group E.

**Conclusion:**

Elevated OSI in the false lumen is associated with a greater tendency toward aortic dilatation following TEVAR for TBAD. Patients with reduced true lumen pressure in the early post-TEVAR period show a corresponding trend toward subsequent aortic dilatation.

## 1 Introduction

Type-B aortic dissection (TBAD) is an aortic disease characterized by a primary tear located distal to the left subclavian artery (Kim et al., 2011). The incidence of TBAD is 1.5 per 100,000 person-years, with a 30-day mortality rate of 13.9% (Obel et al., 2022). Thoracic endovascular aortic repair (TEVAR) has emerged as an important therapeutic modality for TBAD (MacGillivray et al., 2022) and can alter aortic hemodynamics (Karmonik et al., 2011). Since the hemodynamic parameters cannot be directly measured in clinical practice, computational fluid dynamics (CFD) analysis allows for the visualization and quantification of hemodynamics based on three-dimensional (3D) models derived from patient-specific computed tomography angiography (CTA) scans (Morris et al., 2016). Shad et al. (2022) applied CFD simulation and demonstrated that false lumen dilatation after surgical treatment for type-A aortic dissection was associated with blood flow velocity at the tear site. Xu et al. (2018) reported that time-averaged wall shear stress (TAWSS) and relative residence time (RRT) are correlated with false lumen progression in TBAD patients receiving optimal medical treatment.

Although CFD analysis has been widely applied in cardiovascular research, few studies have focused specifically on the role of hemodynamics in aortic dilatation following TEVAR for TBAD. Xu et al. (2017) previously reported that the pressure difference between the true and false lumen and RRT are predictors of false lumen dilatation; however, this study included only two TBAD patients. Therefore, we aimed to investigate the relationship between specific hemodynamic parameters and post-TEVAR aortic dilatation in TBAD patients.

## 2 Materials and methods

### 2.1 Study population

Patients with TBAD who underwent TEVAR at our study center between January 2008 and December 2022 were retrospectively enrolled and screened for eligibility. The dilatation group comprised patients who developed aortic dilatation during post-TEVAR follow-up. The non-dilatation group consisted of patients who did not develop aortic dilatation following TEVAR, who were matched 1:1 with the dilatation group by sex, age, and comorbidities. Control subjects without aortic dissection were selected from a health screening database and matched 1:1 with the dilatation group by sex and age. A patient flowchart detailing enrollment, the inclusion/exclusion criteria, and follow-up is provided in Supplementary Figure S1. To clarify the comparison design, the cohorts were stratified as follows: (1) TBAD with Aortic Dilatation: including Group A (Pre-TEVAR), Group B (1-week post-TEVAR), and Group C (last follow-up); (2) TBAD without Aortic Dilatation: including Group D (Pre-TEVAR) and Group E (1-week post-TEVAR); (3) normal controls: Group F.

### 2.2 Definition

Aortic dilatation on cross-sectional CTA images is defined as meeting either of the following criteria: (i) progressive dilatation: an increase of ≥10 mm per year from the pre-TEVAR baseline to the most recent CTA at the same anatomical level; (ii) absolute diameter: ≥55 mm on the most recent CTA. The total dissection length was measured as the distance from the proximal extent to the distal end of the aortic dissection. The primary intimal tear size was quantified as the maximum diameter on the cross-sectional images. All measurements were performed on high-resolution CTA datasets by two independent investigators who were blinded to patient group assignments, and the average value was used for subsequent analysis to minimize measurement bias.

### 2.3 Follow-up

All patients underwent standardized follow-up CTA at 1, 3, 6, and 12 months post-TEVAR and every 6–12 months thereafter. The pre-TEVAR baseline was defined as the CTA performed within 2 weeks prior to TEVAR. The 1-week post-TEVAR time point was defined as the first available CTA performed within 1 week following the procedure. The 1-week time point was specifically selected because it represents the early postoperative period when the aortic anatomy and hemodynamic environment have stabilized after TEVAR intervention, while avoiding the acute phase (within 24 h–72 h) where residual inflammation, edema, or procedural-related artifacts may interfere with image quality and hemodynamic parameter measurement. This timing allows for an accurate evaluation of the immediate hemodynamic changes after TEVAR and provides a reliable baseline for subsequent long-term follow-up and comparison with the dilatation status. The most recent CTA was defined as the latest CTA scan obtained by the data cutoff date (31 December 2022), which was used to determine the status of aortic dilatation and evaluate the clinical outcomes.

### 2.4 CTA image acquisition

We collected CTA images for the dilatation group pre-TEVAR, 1 week post-TEVAR, and at the most recent follow-up. For the non-dilatation group, we collected CTA images pre-TEVAR and 1 week post-TEVAR. The control group underwent only one CTA scan. All CTA images were acquired as arterial-phase images with significant contrast enhancement.

### 2.5 3D modeling and meshing of the aorta

The CTA images were imported into MIMICS software (Materialise, version 21.0, RRID: SCR_012153) for model reconstruction. Mesh generation was performed using ANSYS Fluent meshing (Ansys Fluent, version 2021, RRID: SCR_022135). The ascending aorta was defined as the flow inlet of the CFD model. The brachiocephalic trunk, left common carotid artery, left subclavian artery, celiac trunk, superior mesenteric artery, bilateral renal arteries, inferior mesenteric artery, and bilateral internal and external iliac arteries served as the outlets. The 3D aortic model was sequentially discretized into surface meshes and volume meshes. The fluid domain was meshed using a poly-hexcore scheme to accurately capture the anatomical geometry of the thoracic aorta. Ten prismatic boundary layers were generated adjacent to the vessel wall to resolve near-wall flow characteristics. Only meshes with a skewness value <0.5 were used for simulations.

In the dilatation group, the 3D models constructed from pre-TEVAR, 1-week post-TEVAR, and most recent follow-up CTA images corresponded to Groups A, B, and C, respectively. In the non-dilatation group, the 3D models constructed from pre-TEVAR and 1-week post-TEVAR CTA images corresponded to Groups D and E, respectively. The normal aortas in Group F served as the control group. The process is shown in Supplementary Figure S2.

### 2.6 Inlet blood flow velocity

Since this was a retrospective study, patient-specific ascending aortic blood flow velocities could not be acquired. Therefore, we measured the ascending aortic blood flow velocities using Doppler ultrasound in three groups of outpatients at our center from January 2023 to October 2023: TBAD patients prior to TEVAR, TBAD patients following TEVAR, and patients with normal aortas. Group-specific average velocity waveforms were constructed for each of the three groups and applied as the inlet boundary conditions for the corresponding CFD models. The waveform amplitude was scaled according to each patient’s body surface area to ensure physiological validity.

### 2.7 Hemodynamic simulation

The mesh models were imported into ANSYS Fluent solver (Ansys Fluent, version 2021, RRID: SCR_022135) to solve the Navier–Stokes equations. This study calculated the following hemodynamic parameters for each model: velocity (m/s), pressure (Pa), wall shear stress (WSS, Pa), TAWSS (Pa), oscillatory shear index (OSI), and RRT. Blood was assumed to be an incompressible, homogeneous Newtonian fluid with a dynamic viscosity of 0.0035 kg/(m·s) and a density of 1,060 kg/m^3^. The aortic wall was assumed to be a rigid wall.

The inlet boundary conditions were derived from the ultrasound-measured velocities in the ascending aorta. The outlet boundary conditions were set as pressure outlets, which were governed by the three-element Windkessel model. The total inlet flow rate was calculated from the Doppler-derived inlet velocity waveform and the inlet cross-sectional area of each model. In the absence of patient-specific branch flow data, the flow was distributed proportionally according to the cross-sectional area of each branch vessel. Based on this distributed flow, the Windkessel resistance and compliance parameters for each outlet were determined to maintain physiological pressure conditions.

All simulations were performed for three complete cardiac cycles with a time step of 0.001 s. A convergence criterion of 1 × 10^−5^ was applied to all residuals to ensure numerical stability. To ensure periodic flow development, only data from the third cardiac cycle were used for post-processing, hemodynamic analysis, and visualization. The velocity, pressure, WSS, TAWSS, OSI, and RRT were visualized using ANSYS CFD-post (Ansys Fluent, version 2021, RRID: SCR_022135).

The values of velocity, pressure, WSS, TAWSS, OSI, and RRT of the vessel wall within the ostia of the major arterial branches, including the brachiocephalic trunk, left common carotid artery, left subclavian artery, primary intimal tear, distal intimal tear, celiac trunk, superior mesenteric artery, bilateral renal arteries, inferior mesenteric artery, and bilateral common iliac arteries, were obtained. In the pre-TEVAR model, the primary intimal tear was defined as the first intimal tear in the proximal segment of the aortic dissection. In the post-TEVAR model, the primary intimal tear was defined as the most proximal intimal tear of the residual dissection through which blood flowed into the false lumen. The distal intimal tear was defined as the most terminal intimal tear of TBAD. The primary CFD endpoint was predefined as the OSI. WSS, TAWSS, and RRT were analyzed as the secondary exploratory hemodynamic parameters to supplement the primary metrics.

### 2.8 Statistical analysis

Continuous data were expressed as the mean ± standard deviation (SD) or median (interquartile range, IQR). Categorical variables were summarized as frequencies and corresponding percentages. Linear mixed-effects models were used to analyze within-subject changes across different time points, accounting for repeated measurements and missing data. Paired t-tests or Wilcoxon signed-rank tests were used for pre–post comparisons where appropriate. To control for multiple testing, the OSI was predefined as the primary endpoint (significance level of *P* < 0.05), and all other outcomes were considered exploratory endpoints. The results were reported as effect sizes with 95% confidence intervals (CIs) and corresponding *P-*values. A two-tailed *P* < 0.05 was considered statistically significant. All statistical analyses were performed using SPSS 25.0 (IBM Corp., Armonk, NY, United States).

## 3 Results

### 3.1 Patient characteristics

A total of 57 participants were enrolled. The proportion of male patients was 78.95% in the dilatation group, 100% in the non-dilatation group, and 73.68% in the control group (*P* = 0.063). The mean ages were 47.63 ± 11.96, 54.26 ± 12.31, and 49.53 ± 12.60 years in the dilatation, non-dilatation, and control groups, respectively (*P* = 0.240). The dilatation and non-dilatation groups were comparable with regard to the procedural characteristics, anatomical features, and follow-up duration. The demographic characteristics of the dilatation and non-dilatation groups are presented in Supplementary Table S1.

### 3.2 Inlet blood flow velocity

We collected the ascending aortic velocities from 10 TBAD patients before TEVAR, 12 TBAD patients after TEVAR, and 19 control subjects. Detailed information is presented in Supplementary Table S2. There was no statistically significant difference in the maximum, minimum, and mean ascending aortic velocities among the three groups (*P* > 0.05). The three group-specific inlet blood flow velocity waveforms are presented in Supplementary Figure S3.

### 3.3 Hemodynamic comparisons

#### 3.3.1 Hemodynamics of Groups A, B, and C

A total of 19 TBAD patients who developed aortic dilatation following TEVAR were enrolled in this study, among whom 12 underwent CTA at 1 week post-TEVAR. Hemodynamic assessment revealed that the pressure values at the ostia of the brachiocephalic trunk, left common carotid artery, left subclavian artery, celiac trunk, superior mesenteric artery, bilateral renal arteries, inferior mesenteric artery, bilateral common iliac arteries, and distal tear in Group B were significantly lower than those observed in Groups A and C. Detailed hemodynamic parameters for Groups A, B, and C are summarized in Supplementary Table S3.

#### 3.3.2 Hemodynamics of Groups D and E

In parallel, 19 TBAD patients without aortic dilatation after TEVAR were also included in the study. Hemodynamic parameters measured before TEVAR (Group D) and at 1 week after TEVAR (Group E) are presented in Supplementary Table S4. Specifically, the OSI at the ostium of the superior mesenteric artery was significantly lower in Group E than in Group D (*P* < 0.05). Furthermore, Group E exhibited higher pressure values than Group D at the ostia of the brachiocephalic trunk, left common carotid artery, left subclavian artery, celiac trunk, superior mesenteric artery, left renal artery, inferior mesenteric artery, and right common iliac artery.

#### 3.3.3 Hemodynamics of Groups A and F

Comparative analysis of the hemodynamic parameters between Group A and the control group (Group F) is provided in Supplementary Table S5. Our findings demonstrated that the RRT at the ostium of the brachiocephalic trunk was significantly lower in Group F than in Group A (*P* = 0.036). Additionally, the velocity, pressure, WSS, and TAWSS at the ostium of the celiac trunk were all significantly lower in Group F than in Group A (all *P* < 0.05). Notably, the pressure at the ostium of the right renal artery was significantly higher in Group A than in Group F (*P* < 0.05).

#### 3.3.4 Hemodynamics of Groups B and F

Hemodynamic comparisons between Groups B and F are illustrated in Supplementary Table S6. The pressure values at the ostia of the brachiocephalic trunk, left common carotid artery, and left subclavian artery were significantly higher in Group B than in Group F (all *P* < 0.05). Moreover, the OSI at the ostium of the right common iliac artery was significantly higher in Group B than in Group F (*P* < 0.05).

#### 3.3.5 Hemodynamics of Groups C and F

Supplementary Table S7 presents the hemodynamic comparisons between Groups C and F. The pressures at the ostia of the brachiocephalic trunk, left common carotid artery, celiac trunk, superior mesenteric artery, and bilateral renal arteries were significantly elevated in Group C relative to Group F (all *P* < 0.05). Additionally, the OSI at the ostium of the left common iliac artery was significantly higher in Group C than in Group F (*P* < 0.05).

#### 3.3.6 Hemodynamics of Groups D and F

Hemodynamic parameters used in comparing Group D and Group F are shown in Supplementary Table S8. The pressure values at the ostia of the left common carotid artery, left subclavian artery, celiac trunk, superior mesenteric artery, inferior mesenteric artery, bilateral renal arteries, and left common iliac artery were significantly lower in Group F than in Group D (all *P* < 0.05).

#### 3.3.7 Hemodynamics of Groups E and F

Supplementary Table S9 displays the hemodynamic parameter comparisons between Groups E and F. The pressures at the ostia of the brachiocephalic trunk, left common carotid artery, left subclavian artery, celiac trunk, superior mesenteric artery, left renal artery, inferior mesenteric artery, left common iliac artery, and right common iliac artery were significantly higher in Group E than in Group F (all *P* < 0.05). In contrast, the OSI at the ostium of the left subclavian artery was significantly lower in Group E than in Group F (*P* = 0.035).

#### 3.3.8 Hemodynamics of Groups A and D

The results of hemodynamic parameter comparisons between Groups A and D are summarized in Supplementary Table S10. Our data indicated that the pressure values at the ostia of the brachiocephalic trunk, left common carotid artery, left subclavian artery, and primary tear were significantly higher in Group A than in Group D.

#### 3.3.9 Hemodynamics of groups B and E

The comparison of hemodynamic parameters of groups B and E is presented in Supplementary Table S11. Specifically, the pressures in Group B at the ostia of the superior mesenteric artery, left renal artery, and right common iliac artery were significantly lower than those in Group E (all *P* < 0.05).

#### 3.3.10 Hemodynamics of Groups C and E

The comparative hemodynamic parameter analysis between Groups C and E is provided in Supplementary Table S12. The pressure values in group C at the ostia of the brachiocephalic trunk, left common carotid artery, left subclavian artery, primary tear, celiac trunk, superior mesenteric artery, bilateral renal arteries, inferior mesenteric artery, bilateral common iliac arteries, and distal tear were all lower than those observed in Group E.

### 3.4 Hemodynamic image illustration

**FIGURE 1:**
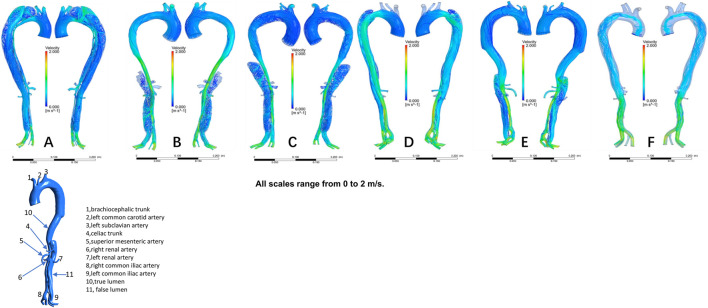
Blood flow patterns and velocity distribution in individual models. **(A)** Pre-TEVAR model in the dilated group. **(B)** 1-week post-TEVAR model in the dilated group. **(C)** Last follow-up model in the dilated group. **(D)** Pre-TEVAR model in the nondilated group. **(E)** 1-week post-TEVAR model in the nondilated group. **(F)** normal aorta model.

**FIGURE 2:**
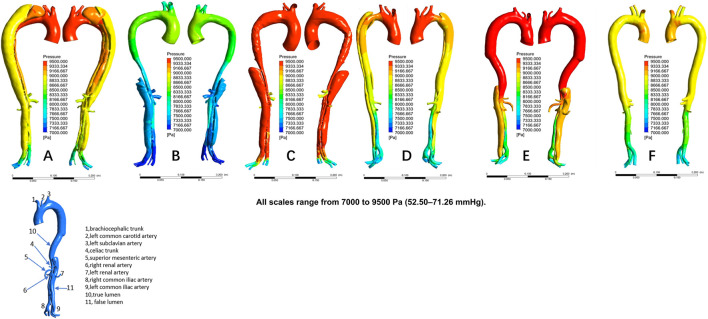
Pressure distribution in individual models. **(A)** Pre-TEVAR model in the dilated group. **(B)** 1-week post-TEVAR model in the dilated group. **(C)** Last follow-up model in the dilated group. **(D)** Pre-TEVAR model in the nondilated group. **(E)** 1-week post-TEVAR model in the nondilated group. **(F)** normal aorta model.

**FIGURE 3:**
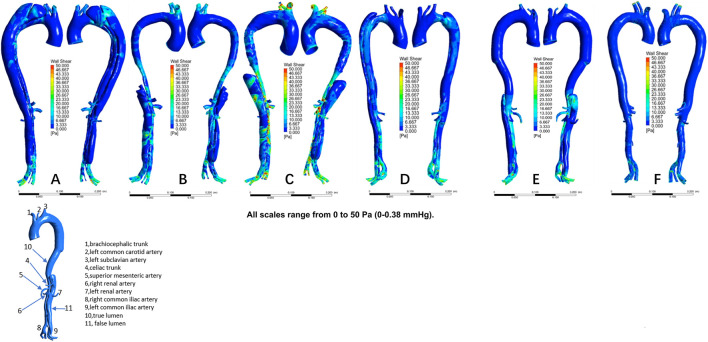
Wall shear stress (WSS) distribution in individual models. **(A)** Pre-TEVAR model in the dilated group. **(B)** 1-week post-TEVAR model in the dilated group. **(C)** Last follow-up model in the dilated group. **(D)** Pre-TEVAR model in the nondilated group. **(E)** 1-week post-TEVAR model in the nondilated group. **(F)** normal aorta model.

**FIGURE 4:**
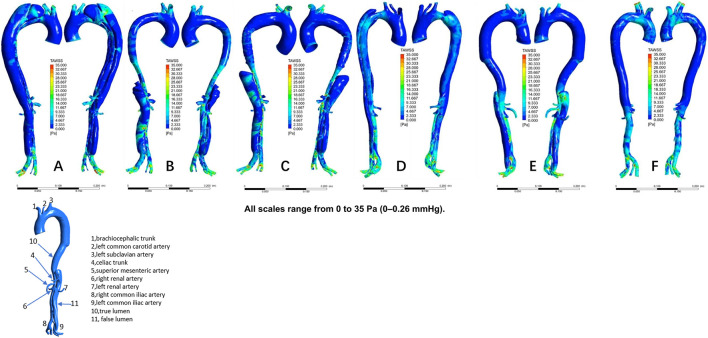
Time-averaged wall shear stress (TAWSS) distribution in individual models. **(A)** Pre-TEVAR model in the dilated group. **(B)** 1-week post-TEVAR model in the dilated group. **(C)** Last follow-up model in the dilated group. **(D)** Pre-TEVAR model in the nondilated group. **(E)** 1-week post-TEVAR model in the nondilated group. **(F)** normal aorta model.

**FIGURE 5:**
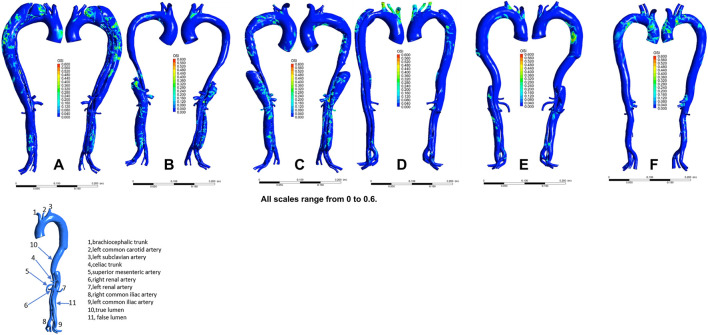
Oscillatory shear index (OSI) distribution in individual models. **(A)** Pre-TEVAR model in the dilated group. **(B)** 1-week post-TEVAR model in the dilated group. **(C)** Last follow-up model in the dilated group. **(D)** Pre-TEVAR model in the nondilated group. **(E)** 1-week post-TEVAR model in the nondilated group. **(F)** normal aorta model.

**FIGURE 6:**
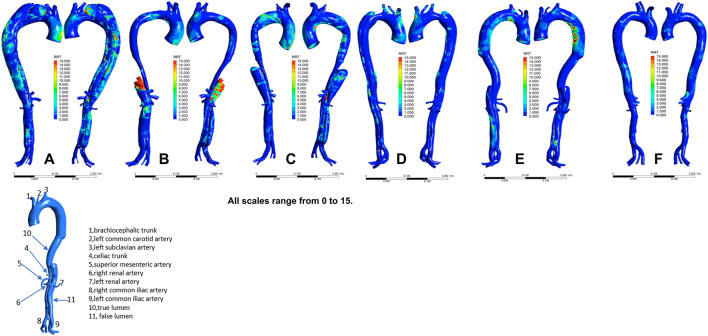
Relative residence time (RRT) distribution in individual models. **(A)** Pre-TEVAR model in the dilated group. **(B)** 1-week post-TEVAR model in the dilated group. **(C)** Last follow-up model in the dilated group. **(D)** Pre-TEVAR model in the nondilated group. **(E)** 1-week post-TEVAR model in the nondilated group. **(F)** normal aorta model.

The distribution of velocities is presented in Figure 1. In the non-dilatation group, the velocity in the true lumen was lower pre-TEVAR than post-TEVAR. In the dilatation group, the most recent follow-up model had a lower velocity in the stent-graft-implanted segment and exhibited more turbulence compared with the 1-week post-TEVAR model. The distribution of pressures is presented in Figure 2. In the non-dilatation group, pressures in the ascending aorta, aortic arch, and thoracic descending aorta were elevated at 1 week post-TEVAR compared with those in the pre-TEVAR model. In the dilatation group, pressures in the ascending aorta, aortic arch, and thoracic descending aorta were lower in the 1-week post-TEVAR model than in the pre-TEVAR model. The distribution of WSS is presented in Figure 3. In the dilatation group, the 1-week post-TEVAR model showed an increase in high-WSS areas compared with the pre-TEVAR model. The distribution of TAWSS is presented in Figure 4. In the non-dilatation group, TEVAR decreased the TAWSS in the thoracic descending aortic segment. In the dilatation group, high-TAWSS areas were most common in the most recent follow-up model (Figure 5C). The distribution of OSI is presented in Figure 5. The false lumen of the pre-TEVAR model in the dilatation group had a larger area of high OSI than that in the non-dilatation group. After TEVAR, the area of high OSI in the false lumen of the non-dilatation group decreased relative to the dilatation group. The distribution of RRT is presented in Figure 6. High RRT was most frequently observed in the false lumen of the pre-TEVAR model in the dilatation group.

## 4 Discussion

Aortic dilatation is an important complication after TEVAR for TBAD, with the prevalence rate ranging from 13.41% to 21.02% (Zhang et al., 2018; Shen et al., 2019). Hemodynamic effects are associated with the development and postoperative regression of TBAD. The increased volume of the proximal aorta causes more aggressive hemodynamics in the distal region of the left subclavian artery, which can lead to TBAD (Peng et al., 2022). Chu et al. (2022) found that in patients with dilated false lumens, the blood flow in the false lumen had higher kinetic energy than that in patients without such dilatation.

We used CFD to analyze the effect of hemodynamics on aortic dilatation after TEVAR for TBAD. We found that in the dilatation group, the pressures at the ostia of the brachiocephalic trunk, left common carotid artery, left subclavian artery, celiac trunk, superior mesenteric artery, bilateral renal arteries, inferior mesenteric artery, bilateral common iliac arteries, and the distal tear decreased at 1 week post-TEVAR compared with those at pre-TEVAR. However, in the non-dilatation group, the pressures at the ostia of the brachiocephalic trunk, left common carotid artery, left subclavian artery, celiac trunk, superior mesenteric artery, left renal artery, inferior mesenteric artery, and right common iliac artery were elevated at 1 week post-TEVAR compared with the pre-TEVAR values. We hypothesize that this characteristic pattern of pressure changes in both groups may reflect hemodynamic associations with aortic dilatation after TEVAR for TBAD. Following TEVAR, endovascular coverage of the primary tear typically restores true lumen blood flow and elevates the intraluminal pressure (Wanhainen et al., 2026). However, in the dilatation group, persistent stenosis of the distal true lumen was observed. This anatomical constraint diverted much of the blood flow into the false lumen, resulting in reduced true lumen perfusion and intraluminal pressure. Consequently, the sustained high-flow state within the false lumen shows an association with progressive aortic dilatation (Chen et al., 2025). Reduced intraluminal pressures can decrease the perfusion pressure of the vasa vasorum in the aortic wall, leading to structural damage and degeneration of the aortic wall (Heistad et al., 1978). The vasa vasorum of the aorta form a capillary network around the adventitia and intima-media of the aortic wall and play a critical role in maintaining the structural and functional integrity of the aortic wall (Osada and Minatoya, 2022). Impairment of their function may lead to hypoxia in aortic wall tissue, impede the transport of cellular nutrients, and ultimately induce medial degeneration of the aortic wall—a major predisposing factor for risk of aortic wall pathological lesions (Osada and Minatoya, 2022).

Elevated WSS can induce structural damage to the aortic vessel wall (Tripathy et al., 2023). We found that the WSS at the ostia of the left renal artery and the brachiocephalic trunk was significantly higher in the dilatation group than in the non-dilatation group at 1 week post-TEVAR. Elevated WSS is correlated with aortic dilatation (Soulat et al., 2022). Zolfaghari et al. (2023) reported that the spatial distribution of elevated WSS was significantly more concentrated in aortas with dilatation than in those without dilatation. Elevated WSS is associated with retrograde type-A aortic dissection following TEVAR for TBAD (Osswald et al., 2017). Reduced WSS is associated with false lumen thrombosis (Munshi et al., 2020). The magnitude and duration of WSS influence the morphology and spatial arrangement of aortic endothelial cells (Goudot and van Kampen, 2023). Under physiological conditions, physiological WSS promotes the production of nitric oxide, which helps maintain normal aortic blood flow and inhibits platelet activation and adhesion (Perinajová et al., 2023). However, elevated WSS induces endothelial dysfunction, platelet activation, upregulation of adhesion molecules, and release of von Willebrand factor, thereby inducing platelet aggregation and thrombus formation (Bańka et al., 2023). Moreover, elevated WSS leads to reduced expression levels of cytoskeletal, actin/myosin, and extracellular matrix proteins, ultimately causing structural dysfunction of the aortic wall tissue (Kobsa et al., 2023).

Elevated OSI is an independent predictor of aortic aneurysm rupture (Lee and Kwak, 2021). Xiao et al. (2023) found that the maximum OSI value was significantly higher in patients with ascending aortic aneurysms than in healthy controls. We observed significantly larger areas of elevated OSI within the false lumen in the dilatation group than in the non-dilatation group. We hypothesize that hemodynamic analysis and OSI distribution show significant associations with false lumen dilatation and may provide clues for clinical evaluation under model assumptions. Liu et al. (2025) demonstrated that regions with elevated OSI are more prone to progression to aortic dissection in patients with intramural hematoma. Elevated OSI directly induces aortic endothelial injury and aortic wall degeneration (Cho, 2023). Human clinical studies have confirmed that following 20 min of induction of a local aortic arterial environment with elevated OSI, the levels of CD62E^+^ endothelial micro-particles increased by approximately fourfold, and those of CD31^+^/CD42b^−^ endothelial micro-particles (a marker of endothelial apoptosis) increased by approximately ninefold (Jenkins et al., 2013).

Elevated RRT is a predictor of thrombus formation (Bäumler et al., 2023). RRT is a hemodynamic parameter that quantifies the relative duration of blood cells residing adjacent to the aortic vessel wall (Jiang et al., 2023). We found a region of elevated RRT in the proximal segment of the false lumen in our CFD model at 1 week post-TEVAR in the dilatation group. We hypothesize that elevated RRT in the proximal segment of the false lumen induces partial false lumen thrombosis, and subsequent partial thrombosis further contributes to negative remodeling of the false lumen. Partial false lumen thrombosis is a key predictor of negative remodeling of the false lumen following TEVAR for TBAD (Gao et al., 2022). Ruiz-Muñoz et al. (2024) suggested that the kinetic energy of blood flow within the false lumen is correlated with the extent of false lumen thrombus formation. Jafarinia et al. (2023) reported that the key predictive parameters for false lumen thrombosis are false lumen diameter and the size and location of the primary intimal tear. A higher risk of partial thrombosis is observed when the false lumen diameter is larger than the true lumen diameter, and a reduction in the ratio of distal to primary intimal tear size increases the risk of persistent false lumen patency (Jafarinia et al., 2023).

### 4.1 Limitations

This study adopted a single-center retrospective design. First, patient-specific intraoperative blood pressure and patient-specific inlet velocity data were unavailable. Generic group-averaged inflow conditions were applied, which cannot fully reflect the true individual hemodynamic status of each patient. Second, blood was assumed to be an incompressible and homogeneous Newtonian fluid under laminar flow conditions, and the aortic wall was defined as rigid, which may neglect the influence of aortic wall elasticity and lead to certain discrepancies in the simulation results. Although numerical stability was verified by a small time step, sufficient cardiac cycles, strict convergence criteria, and mesh independence test, a comprehensive parametric sensitivity analysis was not performed. In addition, the relatively small sample size precluded the need for multivariate regression analysis. Furthermore, all hemodynamic findings related to pressure and OSI represent relative group-level differences and trending characteristics under standardized modeling assumptions rather than absolute indicators that can be used directly for individualized risk stratification.

## 5 Conclusion

Elevated OSI in the false lumen is associated with a greater predisposition for aortic dilatation following TEVAR for TBAD. Patients with lower true lumen pressure in the early post-TEVAR period show a corresponding trend toward subsequent aortic dilatation.

## Data Availability

The original contributions presented in the study are included in the article/Supplementary Material, further inquiries can be directed to the corresponding authors.
