# Supplementary material for: Hemodynamic factors of aortic dilatation after thoracic endovascular aortic repair for type-B aortic dissection
Source: Front Bioeng Biotechnol. 2026 Apr 22;14:1780047. doi: 10.3389/fbioe.2026.1780047 (PMC13143993; doi:10.3389/fbioe.2026.1780047)
Supplement: Supplementary file 1 [file Table1.docx]

Supplementary Table 1 Demographic characteristics of the dilated and nondilated groups

| Variable | Dilated group (n=19) | Nondilated group (n=19) | OR/MD (95% CI) | P value |
| --- | --- | --- | --- | --- |
| Weight (kg) | 72.21±10.98 | 73.61±12.89 | 14.44(-9.73,6.94) | 0.339 |
| Height (cm) | 168.41±6.03 | 169.65±4.91 | 8.84(-5.78,3.31) | 0.244 |
| Systolic blood pressure (mmHg) | 144.74±32.22 | 146.95±28.64 | 36.02(-19.57,15.15) | 0.206 |
| Diastolic blood pressure (mmHg) | 88.42±22.16 | 86.37±19.97 | 23.89(-9.46,13.57) | 0.129 |
| Chest pain | 8(42.11) | 13(68.42) | 0.34(0.09,1.27) | 0.103 |
| Chest tightness | 3(15.79) | 1(5.26) | 3.38(0.32,35.79) | 0.290 |
| Abdominal pain | 6(31.58) | 8(42.11) | 0.64(0.17,2.40) | 0.501 |
| Back pain | 8(42.11) | 6(31.59) | 1.58(0.42,5.95) | 0.501 |
| Coronary artery disease | 1(5.26) | 2(10.53) | 0.47(0.04,5.70) | 0.547 |
| Stroke | 3(15.79) | 1(5.26) | 3.38(0.32,35.79) | 0.290 |
| Renal insufficient | 1(2.26) | 3(15.79) | 0.30(0.03,3.14) | 0.290 |
| Hypertension | 12(63.16) | 17(89.47) | 0.20(0.04,1.15) | 0.056 |
| Diabetes | 0 | 2(10.53) | 0.90(0.77,1.04) | 0.146 |
| Marfan syndrome | 3(15.79) | 0 | 1.19(0.98,1.44) | 0.071 |
| Autoimmune disease | 2(10.53) | 3(15.79) | 0.63(0.09,4.26) | 0.631 |
| History of other surgery | 3(15.79) | 4(21.05) | 0.70(0.13,3.68) | 0.676 |
| Smoking | 8(42.11) | 7(36.84) | 1.25(0.34,4.59) | 0.740 |
| Alcohol intake | 8(42.11) | 10(52.63) | 0.66(0.18,2.36) | 0.516 |
| Operation time (min) | 176.21±122.00 | 128.42±103.74 | 174.86(-36.49,132.07) | 0.424 |
| Length of stent graft coverage (mm) | 181.58±55.90 | 174.21±35.52 | 52.50(-17.94,32.67) | 0.081 |
| Hybrid operation | 1(5.26) | 1(5.26) | 1.00(0.06,17.25) | 1.000 |
| Coverage of LSA | 6(31.58) | 8(42.11) | 0.64(0.17,2.40) | 0.501 |
| Reconstruction of LSA | 2(10.53) | 2(10.53) | 1.00(0.13,7.94) | 1.000 |
| Number of intimal tears | 4.84±2.59 | 4.58±2.39 | 3.48(-1.41,1.94) | 0.920 |
| Primary tear size (mm) | 8.41±3.04 | 12.14±8.81 | 8.84(-7.99,0.53) | 0.508 |
| Distance from primary tear to LSA (mm) | 28.90(10.21,61.94) | 19.05±11.88 | 53.42(-3.91,47.59) | 0.561 |
| Length of dissection(mm) | 372.74±63.65 | 393.03±67.16 | 103.12(-69.99,29.41) | 0.318 |
| Follow-up period (months) | 41.33(6.41,69.62) | 30.68(3.22,93.70) | 52.83(-31.32,19.61) | 0.292 |
| Complicated TBAD | 6(31.58) | 3(15.79) | 2.46(0.51,11.80) | 0.252 |
| Partially thrombosed false lumen | 11(57.89) | 9(47.37) | 1.53(0.42,5.50) | 0.516 |

LSA, left subclavian artery. TBAD, type B aortic dissection. OR, odds ratio. MD, mean difference.95% CI, 95% confidence interval. Continuous data were expressed as mean ± standard deviation or median and interquartile range. Categorical variables were expressed as absolute values and percentages.
