# Supplementary material for: Hemodynamic factors of aortic dilatation after thoracic endovascular aortic repair for type-B aortic dissection
Source: Front Bioeng Biotechnol. 2026 Apr 22;14:1780047. doi: 10.3389/fbioe.2026.1780047 (PMC13143993; doi:10.3389/fbioe.2026.1780047)
Supplement: Supplementary file 2 [file Table8.docx]

Supplementary Table 8 Pre-TEVAR hemodynamics in the nondilated group versus the control group

| Location | Variable | Group D(n=19) | Group F(n=19) | MD (95% CI) | P value |
| --- | --- | --- | --- | --- | --- |
| BCT | Velocity | 0.02(0.01,0.05) | 0.09(0.05,0.13) | 0.05(0.02,0.11) | 0.002 |
|  | Pressure | 8893.38±1487.33 | 7599.91±356.60 | -1293.47(-2051.18,-535.76) | 0.002 |
|  | WSS | 1.11(0.48,1.63) | 4.17(1.15,5.31) | 2.89(1.63,3.44) | 0.002 |
|  | TAWSS | 1.29(0.51,2.36) | 4.25(1.55,4.65) | 2.44(1.01,3.32) | 0.001 |
|  | OSI | 0.01(0.001,0.13) | 0.002(0,0.04) | -0.003(-0.08,0.01) | 0.306 |
|  | RRT | 0.85(0.43,2.24) | 0.25(0.22,1.09) | -0.38(-0.93,-0.18) | 0.014 |
| LCCA | Velocity | 0.03(0.02,0.05) | 0.03(0.02,0.13) | -0.003(-0.03,0.02) | 0.717 |
|  | Pressure | 8904.63±1450.95 | 7569.82±356.22 | -1334.81(-2072.72,-596.91) | 0.001 |
|  | WSS | 0.92(0.29,1.90) | 0.99(0.81,3.40) | 0.66(-0.29,1.96) | 0.277 |
|  | TAWSS | 1.10(0.41,2.81) | 1.74(0.80,2.53) | 0.34(-0.92,1.32) | 0.445 |
|  | OSI | 0.01(0.004,0.03) | 0.01(0.002,0.04) | -0.001(-0.01,0.02) | 0.872 |
|  | RRT | 0.92(0.45,2.52) | 0.62(0.41,1.66) | -0.14(-1.45,0.71) | 0.421 |
| LSA | Velocity | 0.02(0.02,0.05) | 0.04(0.04,0.06) | 0.02(-0.01,0.03) | 0.205 |
|  | Pressure | 8879.44±1418.32 | 7550.29±358.58 | -1329.15(-2053.77,-604.53) | 0.001 |
|  | WSS | 1.30(0.64,3.39) | 1.54(0.59,2.81) | 0.26(-2.10,1.78) | 0.809 |
|  | TAWSS | 1.64(0.84,3.09) | 1.24(0.90,3.79) | 0.16(-1010,1.51) | 0.778 |
|  | OSI | 0.01(0.001,0.10) | 0.04(0.01,0.17) | 0.01(-0.01,0.10) | 0.376 |
|  | RRT | 0.73(0.37,1.34) | 0.92(0.29,1.83) | -0.13(-0.56,0.86) | 0.872 |
| Celiac trunk | Velocity | 0.09(0.04,0.41) | 0.03(0.03,0.11) | -0.06(-0.24,-0.02) | 0.005 |
|  | Pressure | 8463.65±653.91 | 7510.09±360.37 | -953.56(-1347.76,-559.35) | 0.000 |
|  | WSS | 2.37(1.15,14.86) | 1.08(0.43,2.46) | -1.48(-11.32,-0.62) | 0.001 |
|  | TAWSS | 3.07(1.98,15.41) | 1.33(0.34,2.27) | -2.64(-9.73,-1.25) | 0.001 |
|  | OSI | 0.001(0,0.01) | 0.001(0,0.02) | 0.00(-0.001,0.002) | 0.777 |
|  | RRT | 0.33(0.07,0.51) | 0.76(0.44,3.05) | 0.43(0.22,0.81) | 0.049 |
| SMA | Velocity | 0.08(0.06,0.18) | 0.03(0.02,0.11) | -0.04(-0.10,-0.003) | 0.039 |
|  | Pressure | 8498.41±785.94 | 7509.93±370.65 | -988.48(-1445.24,-531.72) | 0.000 |
|  | WSS | 2.78(1.49,8.29) | 1.27(0.44,2.95) | -1.28(-4.80,0.05) | 0.108 |
|  | TAWSS | 2.27(1.65,10.54) | 1.12(0.50,2.57) | -1.57(-6.86,-0.37) | 0.006 |
|  | OSI | 0.01(0,0.04) | 0.003(0.001,0.02) | 0.001(-0.01,0.004) | 0.660 |
|  | RRT | 0.44(0.10,0.61) | 0.93(0.39,1.99) | 0.43(0.17,0.92) | 0.010 |
| LRA | Velocity | 0.096(0.050,0.218) | 0.04(0.03,0.07) | -0.02(-0.11,-0.001) | 0.050 |
|  | Pressure | 8645.17±659.40 | 7498.38±382.51 | -1146.79(-1560.47,-733.11) | 0.001 |
|  | WSS | 2.57(1.38,6.17) | 2.04(1.05,3.80) | -0.50(-2.91,1.63) | 0.355 |
|  | TAWSS | 2.79(1.90,6.75) | 3.01(1.16,3.96) | -0.47(-3.29,1.47) | 0.334 |
|  | OSI | 0.01(0.001,0.04) | 0.001(0.001,0.01) | -0.001(-0.02,0.001) | 0.155 |
|  | RRT | 0.39(0.17,0.59) | 0.48(0.27,0.88) | 0.11(-0.34,0.36) | 0.717 |
| RRA | Velocity | 0.04(0.02,0.16) | 0.04(0.02,0.06) | -0.002(-0.06,0.01) | 0.398 |
|  | Pressure | 8633.09±698.17 | 7501.04±371.53 | -1132.04(-1564.08,-700.01) | 0.001 |
|  | WSS | 3.98(1.08,6.17) | 2.37(1.57,4.20) | -0.37(-3.22,1.14) | 0.295 |
|  | TAWSS | 3.97(1.85,6.51) | 2.67(1.61,4.51) | -1.16(-4.40,2.16) | 0.227 |
|  | OSI | 0.001(0,0.003) | 0(0,0.003) | 0.00(-0.001,0.001) | 0.360 |
|  | RRT | 0.27(0.15,0.63) | 0.38(0.22,0.62) | 0.10(-0.23,0.29) | 0.601 |
| IMA | Velocity | 0.06(0.02,0.24) | 0.03(0.02,0.04) | -0.03(-0.20,0.003) | 0.059 |
|  | Pressure | 8301.16±584.83 | 7424.77±430.94 | -860.20(-1270.32,-450.07) | 0.001 |
|  | WSS | 5.62(2.75,14.05) | 1.24(0.77,2.70) | -3.91(-9.81,-1.19) | 0.002 |
|  | TAWSS | 6.89(2.54,9.46) | 1.72(0.77,2.70) | -3.99(-7.13,-1.56) | 0.002 |
|  | OSI | 0(0,0.02) | 0.002(0,0.011) | 0.00(-0.01.01) | 0.842 |
|  | RRT | 0.15(0.11,0.44) | 0.59(0.37,1.31) | 0.34(0.05,1.08) | 0.059 |
| LCIA | Velocity | 0.10(0.02,0.15) | 0.16(0.06,0.31) | 0.09(-0.05,0.18) | 0.091 |
|  | Pressure | 8306.75±784.46 | 7344.30±488.06 | -962.46(-1472.11,-452.81) | 0.001 |
|  | WSS | 9.39(2.34,15.97) | 7.53(3.97,11.24) | -0.38(-5.55,4.44) | 0.629 |
|  | TAWSS | 9.49(2.06,14.15) | 7.67(4.21,11.40) | -1.42(-5.29,7.25) | 0.968 |
|  | OSI | 0.001(0,0.003) | 0(0,0.001) | 0.00(-0.002,0.00) | 0.600 |
|  | RRT | 0.11(0.07,0.49) | 0.13(0.09,0.24) | 0.01(-.36,0.13) | 0.687 |
| RCIA | Velocity | 0.06(0.02,0.13) | 0.15(0.04,0.30) | 0.07(-0.06,0.21) | 0.212 |
|  | Pressure | 7908.17±2031.96 | 7375.50±439.83 | -532.67(-1574.52,509.17) | 0.297 |
|  | WSS | 8.83(3.86,15.01) | 6.63(2.81,10.25) | -2.33(-6.36,2.45) | 0.284 |
|  | TAWSS | 8.49(3.68,13.31) | 6.17(2.65,11.14) | -1.26(-8.10,4.15) | 0.469 |
|  | OSI | 0.001(0,0.002) | 0(0,0.001) | 0.00(-0.002,0.00) | 0.023 |
|  | RRT | 0.12(0.08,0.27) | 0.16(0.09,0.38) | 0.04(-0.04,0.12) | 0.546 |

Group D: Pre-TEVAR hemodynamics in the nondilated group. Group F: Normal control group. TEVAR, thoracic endovascular aortic repair. MD, Median difference.95% CI, 95% confidence interval. BCT, brachiocephalic trunk; LCCA, left common carotid artery; LSA, left subclavian artery; SMA, superior mesenteric artery; LRA, left renal artery; RRA, right renal artery; IMA, inferior mesenteric artery; LCIA, left common iliac artery; RCIA, right common iliac artery. WSS, wall shear stress; TAWSS, time-averaged wall shear stress; OSI, oscillatory shear index; RRT, relative residence time. Velocity is presented in m/s, pressure in Pa, and WSS in Pa. Continuous data were expressed as mean ± standard deviation or median and interquartile range. Categorical variables were reported as absolute values and percentages.
