# Supplementary material for: Hemodynamic factors of aortic dilatation after thoracic endovascular aortic repair for type-B aortic dissection
Source: Front Bioeng Biotechnol. 2026 Apr 22;14:1780047. doi: 10.3389/fbioe.2026.1780047 (PMC13143993; doi:10.3389/fbioe.2026.1780047)
Supplement: Supplementary file 4 [file Table12.docx]

**Supplementary Table 12 Comparison of hemodynamics between the dilated group at last follow-up and the nondilated group at 1-week post-TEVAR**

| Location | Variable | Group C(n=19) | Group E(n=19) | MD (95% CI) | P value |
| --- | --- | --- | --- | --- | --- |
| BCT | Velocity | 0.05(0.04,0.09) | 0.03(0.01,0.07) | -0.03(-0.05,0.02) | 0.167 |
|  | Pressure | 9513.99±3534.00 | 14164.99±12553.67 | 4650.99(-1901.49,11203.48) | 0.153 |
|  | WSS | 2.17(0.84,7.76) | 1.28(0.39,2.10) | -0.81(-2.66,0.46) | 0.359 |
|  | TAWSS | 1.76(0.79,4.31) | 1.48(0.28,2.58) | -0.93(-2.76,0.11) | 0.126 |
|  | OSI | 0.001(0,0.092) | 0.01(0,0.04) | 0.00(-0.05,0.01) | 0.583 |
|  | RRT | 0.59(0.23,2.14) | 0.68(0.39,1.35) | 0.27(-0.35,0.88) | 0.648 |
| LCCA | Velocity | 0.04(0.02,0.09) | 0.04(0.02,0.10) | 0.01(-0.06,0.03) | 0.717 |
|  | Pressure | 9538.10±3122.78 | 13988.33±12150.58 | 4450.23(-1860.27,10760.72) | 0.156 |
|  | WSS | 1.23(0.69,5.27) | 1.16(0.51,2.56) | -0.60(-2.14,1.07) | 0.359 |
|  | TAWSS | 1.64(0.63,3.35) | 1.53(0.62,2.70) | -0.59(-1.93,1.08) | 0.469 |
|  | OSI | 0.002(0,0.057) | 0.01(0,0.04) | 0.002(-0.002,0.04) | 0.629 |
|  | RRT | 0.61(0.30,1.59) | 0.71(0.37,4.08) | 0.47(-0.96,2.93) | 0.277 |
| LSA | Velocity | 0.07(0.04,0.14) | 0.05(0.02,0.09) | -0.02(-0.07,0.02) | 0.332 |
|  | Pressure | 9892.98±4589.86 | 13908.68±12055.39 | 5090.41(-2147.78,12328.60) | 0.155 |
|  | WSS | 1.91(0.56,8.55) | 1.20(0.48,2.12) | -0.65(-3.41,0.14) | 0.143 |
|  | TAWSS | 1.20(0.95,6.83) | 1.66(0.58,2.64) | -0.44(-4.11,-0.10) | 0.049 |
|  | OSI | 0.002(0,0.067) | 0.02(0,0.03) | 0.00(-0.01,0.02) | 0.791 |
|  | RRT | 0.91(0.15,1.24) | 0.63(0.39,2.11) | 3.14(-6.37,12.66) | 0.494 |
| Primary tear | Velocity | 0.08(0.06,0.29) | 0.17(0.08,0.34) | 0.01(-0.05,0.13) | 0.523 |
|  | Pressure | 8786.91±2241.98 | 9185.82±1878.48 | 271.45(-1446.43,1989.32) | 0.742 |
|  | WSS | 3.61(1.56,18.37) | 5.81(0.64,21.34) | 2.05(-3.57,5.71) | **0.629** |
|  | TAWSS | 3.31(1.68,19.31) | 5.21(0.65,19.79) | 1.93(-2.62,5.53) | 0.492 |
|  | OSI | 0(0,0.004) | 0.001(0,0.017) | 0.00(-0.002,0.01) | 0.791 |
|  | RRT | 0.44(0.05,0.61) | 0.20(0.05,1.73) | -0.01(-0.44,0.56) | **0.925** |
| Celiac trunk | Velocity | 0.13(0.04,0.25) | 0.24(0.07,0.33) | 0.03(-0.04,0.20) | 0.167 |
|  | Pressure | 8113.18±973.74 | 8920.65±1405.44 | 807.47(-43.24,1658.17) | 0.062 |
|  | WSS | 7.72(2.00,14.60) | 4.82(1.34,8.83) | -5.05(-7.20,-0.08) | 0.064 |
|  | TAWSS | 6.48(3.01,14.67) | 4.57(2.26,8.51) | -2.76(-6.16,-0.83) | 0.039 |
|  | OSI | 0.002(0,0.017) | 0.002(0,0.02) | 0.00(-0.002,0.005) | 0.910 |
|  | RRT | 0.19(0.07,0.33) | 0.23(0.12,0.45) | 0.06(0.01,0.24) | 0.033 |
| SMA | Velocity | 0.07(0.04,0.27) | 0.18(0.09,0.42) | 0.13(0.03,0.18) | 0.039 |
|  | Pressure | 8226.23±968.64 | 9062.77±1390.25 | 836.54(-65.10,1738.18) | 0.067 |
|  | WSS | 3.93(0.99,12.54) | 6.42(1.99,11.06) | 0.40(-2.07,5.08) | 0.776 |
|  | TAWSS | 3.71(1.01,11.24) | 8.11(1.20,10.50) | -0.06(-3.06,7.26) | 0.520 |
|  | OSI | 0(0,0.003) | 0.001(0,0.003) | 0.00(-0.001,0.002) | 0.607 |
|  | RRT | 0.28(0.09,1.29) | 0.12(0.10,0.84) | 0.02(-0.21,0.07) | 0.809 |
| LRA | Velocity | 0.06(0.02,0.18) | 0.066(0.028,0.152) | 0.01(-0.11,0.05) | 0.702 |
|  | Pressure | 8482.31±1637.24 | 9055.07±1334.25 | 572.77(-496.65,1642.18) | 0.275 |
|  | WSS | 5.19(1.09,10.11) | 2.17(0.43,6.82) | -1.28(-3.15,0.46) | 0.648 |
|  | TAWSS | 4.26(1.22,9.85) | 2.30(0.37,6.79) | -0.21(-3.35,1.89) | 0.687 |
|  | OSI | 0.002(0,0.008) | 0.02(0.001,0.05) | 0.01(-0.002,0.04) | 0.332 |
|  | RRT | 0.24(0.10,0.83) | 0.46(0.15,2.69) | 0.05(-0.40,0.37) | 0.717 |
| RRA | Velocity | 0.035(0.021,0.108) | 0.09(0.02,0.12) | 0.03(-0.04,0.05) | 0.520 |
|  | Pressure | 8350.595±1057.603 | 8549.70±2429.54 | 199.11(-986.45,1384.66) | 0.728 |
|  | WSS | 6.636(4.072,10.101) | 3.64(1.25,10.99) | -1.53(-8.70,3.45) | 0.546 |
|  | TAWSS | 6.271(2.975,9.504) | 4.37(0.80,7.96) | -0.17(-7.92,2.47) | 0.355 |
|  | OSI | 0.001(0,0.005) | 0.005(0.002,0.02) | 0.004(-0.002,0.02) | 0.167 |
|  | RRT | 0.17(0.11,0.34) | 0.23(0.13,1.25) | 0.04(-0.14,0.25) | 0.494 |
| IMA | Velocity | 0.02(0.01,0.05) | 0.05(0.02,0.16) | 0.01(-0.02,0.13) | 0.394 |
|  | Pressure | 7736.95±621.29 | 8435.11±1133.46 | 1053.06(339.56,1766.55) | 0.007 |
|  | WSS | 3.31(1.40,15.81) | 3.55(1.18,15.34) | -0.57(-4.79,1.26) | 0.307 |
|  | TAWSS | 2.27(1.87,8.27) | 3.40(0.67,6.92) | -1.42(-4.29,0.50) | 0.118 |
|  | OSI | 0.001(0,0.010) | 0.003(0,0.01) | 0.00(-0.004,0.002) | 0.666 |
|  | RRT | 0.44(0.12,0.57) | 0.30(0.150,1.54) | 0.13(0.02,1.11) | 0.071 |
| LCIA | Velocity | 0.05(0.02,0.14) | 0.12(0.05,0.37) | 0.07(0.004,0.11) | 0.039 |
|  | Pressure | 7888.64±1129.63 | 8229.17±897.48 | 340.53(-343.75,1024.81) | 0.310 |
|  | WSS | 4.74(3.50,8.10) | 9.68(2.19,21.69) | 2.89(-2.33,15.43) | 0.648 |
|  | TAWSS | 5.96(4.05,8.26) | 10.00(2.14,18.60) | 5.09(-3.98,12.60) | 0.260 |
|  | OSI | 0.001(0,0.012) | 0.001(0,0.005) | 0.00(-0.01,0.002) | 0.529 |
|  | RRT | 0.18(0.12,0.25) | 0.10(0.05,0.47) | -0.05(-0.14,0.07) | 0.872 |
| RCIA | Velocity | 0.03(0.02,0.19) | 0.13(0.07,0.22) | 0.06(0.01,0.13) | 0.033 |
|  | Pressure | 7933.93±1428.34 | 8170.14±843.71 | 236.22(-587.90,1060.33) | 0.555 |
|  | WSS | 4.79(2.34,14.37) | 7.63(3.39,13.84) | -0.99(-7.12,4.88) | 0.819 |
|  | TAWSS | 4.95(2.48,16.06) | 6.97(2.03,12.68) | -1.14(-9.93,4.06) | 0.601 |
|  | OSI | 0.001(0,0.003) | 0.001(0,0.006) | 0.00(-0.002,0.001) | 0.834 |
|  | RRT | 0.20(0.06,0.41) | 0.14(0.08,0.50) | 0.04(-0.15,0.09) | 0.648 |
| Distal tear | Velocity | 0.07(0.03,0.12) | 0.12(0.03,0.17) | 0.02(-0.03,0.07) | 0.815 |
|  | Pressure | 7684.53±923.51 | 7993.32±1109.31 | 181.51(-463.19,826.20) | 0.560 |
|  | WSS | 5.30(0.58,8.84) | 5.71(1.49,21.94) | 1.88(0.34,8.72) | 0.048 |
|  | TAWSS | 5.27(0.55,9.44) | 5.83(1.77,21.48) | 0.97(-0.09,6.90) | 0.238 |
|  | OSI | 0.005(0,0.083) | 0.002(0,0.01) | 0.00(-0.01,0.01) | 0.679 |
|  | RRT | 0.21(0.11,1.85) | 0.19(0.05,0.60) | -0.02(-0.70,0.01) | 0.332 |

Group C: Hemodynamics at the last follow‑up in the dilated group. Group E: Hemodynamics at 1-week post-TEVAR in the nondilated group. TEVAR, thoracic endovascular aortic repair. MD, Median difference.95% CI, 95% confidence interval. BCT, brachiocephalic trunk; LCCA, left common carotid artery; LSA, left subclavian artery; SMA, superior mesenteric artery; LRA, left renal artery; RRA, right renal artery; IMA, inferior mesenteric artery; LCIA, left common iliac artery; RCIA, right common iliac artery. WSS, wall shear stress; TAWSS, time-averaged wall shear stress; OSI, oscillatory shear index; RRT, relative residence time. Velocity is presented in m/s, pressure in Pa, and WSS in Pa. Continuous data were expressed as mean ± standard deviation or median and interquartile range. Categorical variables were reported as absolute values and percentages.
