# Supplementary material for: Hemodynamic factors of aortic dilatation after thoracic endovascular aortic repair for type-B aortic dissection
Source: Front Bioeng Biotechnol. 2026 Apr 22;14:1780047. doi: 10.3389/fbioe.2026.1780047 (PMC13143993; doi:10.3389/fbioe.2026.1780047)
Supplement: Supplementary file 6 [file Table7.docx]

**Supplementary Table 7 Comparison of hemodynamics at last follow-up between the dilated group and the control group**

| Location | Variable | Group C(n=19) | Group F(n=19) | MD (95% CI) | P value |
| --- | --- | --- | --- | --- | --- |
| BCT | Velocity | 0.05(0.04,0.09) | 0.09(0.05,0.13) | 0.001(-0.04,0.06) | 0.546 |
|  | Pressure | 9513.99±3534.00 | 7599.91±356.60 | 1914.09(170.07,3658.11) | 0.033 |
|  | WSS | 2.17(0.84,7.76) | 4.17(1.15,5.31) | 0.60(-2.21,3.41) | 0.809 |
|  | TAWSS | 1.76(0.79,4.31) | 4.25(1.55,4.65) | 0.34(-2.30,3.27) | 0.573 |
|  | OSI | 0.001(0,0.092) | 0.002(0,0.04) | 0.00(-0.03,0.02) | 0.717 |
|  | RRT | 0.59(0.23,2.14) | 0.25(0.22,1.09) | -0.14(-1.03,0.13) | 0.099 |
| LCCA | Velocity | 0.04(0.02,0.09) | 0.03(0.02,0.13) | -0.002(-0.05,0.03) | 0.809 |
|  | Pressure | 9538.10±3122.78 | 7569.82±356.22 | -1968.28(-3508.90,-427.66) | 0.015 |
|  | WSS | 1.23(0.69,5.27) | 0.99(0.81,3.40) | -0.11(-1.63,0.70) | 0.601 |
|  | TAWSS | 1.64(0.63,3.35) | 1.74(0.80,2.53) | -0.20(-1.67,1.61) | 0.889 |
|  | OSI | 0.002(0,0.057) | 0.01(0.002,0.04) | 0.00(0.00,0.03) | 0.875 |
|  | RRT | 0.61(0.30,1.59) | 0.62(0.41,1.66) | -0.08(-1.17,0.43) | 0.629 |
| LSA | Velocity | 0.07(0.04,0.14) | 0.04(0.04,0.06) | -0.01(-0.10,0.04) | 0.381 |
|  | Pressure | 9892.98±4589.86 | 7550.29±358.58 | -1745.12(-4371.51,863.28) | 0.175 |
|  | WSS | 1.91(0.56,8.55) | 1.54(0.59,2.81) | -0.04(-4.58,0.98) | 0.407 |
|  | TAWSS | 1.20(0.95,6.83) | 1.24(0.90,3.79) | -0.48(3.23,1.07) | 0.554 |
|  | OSI | 0.002(0,0.067) | 0.04(0.01,0.17) | 0.02(-0.01,0.15) | 0.080 |
|  | RRT | 0.91(0.15,1.24) | 0.92(0.29,1.83) | 0.09(-0.44,1.16) | 0.407 |
| Celiac trunk | Velocity | 0.13(0.04,0.25) | 0.03(0.03,0.11) | -0.03(-0.12,0.002) | 0.108 |
|  | Pressure | 8113.18±973.74 | 7510.09±360.37 | -603.09(-1113.26,-92.93) | 0.023 |
|  | WSS | 7.72(2.00,14.60) | 1.08(0.43,2.46) | -6.59(-12.39,-1.45) | 0.000 |
|  | TAWSS | 6.48(3.01,14.67) | 1.33(0.34,2.27) | -5.24(-10.71,-2.72) | 0.000 |
|  | OSI | 0.002(0,0.017) | 0.001(0,0.02) | 0.00(-0.002,0.001) | 0.876 |
|  | RRT | 0.19(0.07,0.33) | 0.76(0.44,3.05) | 0.39(0.26,1.21) | 0.011 |
| SMA | Velocity | 0.07(0.04,0.27) | 0.03(0.02,0.11) | -0.05(-0.16,-0.02) | 0.036 |
|  | Pressure | 8226.23±968.64 | 7509.93±370.65 | -716.31(-1225.88,-206.73) | 0.009 |
|  | WSS | 3.93(0.99,12.54) | 1.27(0.44,2.95) | -2.43(-9.24,-0.42) | 0.006 |
|  | TAWSS | 3.71(1.01,11.24) | 1.12(0.50,2.57) | -1.80(-6.32,0.26) | 0.011 |
|  | OSI | 0(0,0.003) | 0.003(0.001,0.02) | 0.001(0.00,0.01) | 0.139 |
|  | RRT | 0.28(0.09,1.29) | 0.93(0.39,1.99) | 0.28(-0.04,0.99) | 0.184 |
| LRA | Velocity | 0.06(0.02,0.18) | 0.04(0.03,0.07) | -0.01(-0.14,0.02) | 0.295 |
|  | Pressure | 8482.31±1637.24 | 7498.38±382.51 | -983.93(-1792.22,-175.63) | 0.020 |
|  | WSS | 5.19(1.09,10.11) | 2.04(1.05,3.80) | -2.71(-4.94,2.24) | 0.077 |
|  | TAWSS | 4.26(1.22,9.85) | 3.01(1.16,3.96) | -1.53(-5.14,2.27) | 0.136 |
|  | OSI | 0.002(0,0.008) | 0.001(0.001,0.01) | 0.001(0.00,0.01) | 0.300 |
|  | RRT | 0.24(0.10,0.83) | 0.48(0.27,0.88) | 0.15(-0.23,0.47) | 0.520 |
| RRA | Velocity | 0.035(0.021,0.108) | 0.04(0.02,0.06) | -0.004(-0.04,0.02) | 0.557 |
|  | Pressure | 8350.595±1057.603 | 7501.04±371.53 | -849.55(-1395.23,-303.88) | 0.004 |
|  | WSS | 6.636(4.072,10.101) | 2.37(1.57,4.20) | -4.48(-7.92,-1.55) | 0.003 |
|  | TAWSS | 6.271(2.975,9.504) | 2.67(1.61,4.51) | -3.43(-7.33,-0.78) | 0.008 |
|  | OSI | 0.001(0,0.005) | 0(0,0.003) | 0.00(-0.002,0.002) | 0.637 |
|  | RRT | 0.17(0.11,0.34) | 0.38(0.22,0.62) | 0.20(0.04,0.30) | 0.039 |
| IMA | Velocity | 0.02(0.01,0.05) | 0.03(0.02,0.04) | 0.004(-0.01,0.01) | 0.593 |
|  | Pressure | 7736.95±621.29 | 7424.77±430.94 | -282.77(-846.91,281.36) | 0.281 |
|  | WSS | 3.31(1.40,15.81) | 1.24(0.77,2.70) | -1.34(-4.59,0.87) | 0.088 |
|  | TAWSS | 2.27(1.87,8.27) | 1.72(0.77,2.70) | -0.60(-4.89,0.11) | 0.197 |
|  | OSI | 0.001(0,0.010) | 0.002(0,0.011) | 0.00(-0.01,0.01) | 0.944 |
|  | RRT | 0.44(0.12,0.57) | 0.59(0.37,1.31) | 0.20(-.0.06,0.48) | 0.100 |
| LCIA | Velocity | 0.05(0.02,0.14) | 0.16(0.06,0.31) | 0.12(0.001,0.17) | 0.033 |
|  | Pressure | 7888.64±1129.63 | 7344.30±488.06 | -544.35(-1121.80,33.11) | 0.063 |
|  | WSS | 4.74(3.50,8.10) | 7.53(3.97,11.24) | 3.45(-0.76,5.10) | 0.398 |
|  | TAWSS | 5.96(4.05,8.26) | 7.67(4.21,11.40) | 1.67(-1.35,5.22) | 0.494 |
|  | OSI | 0.001(0,0.012) | 0(0,0.001) | -0.001(-0.01,0.00) | 0.036 |
|  | RRT | 0.18(0.12,0.25) | 0.13(0.09,0.24) | -.0.04(-0.11,0.06) | 0.469 |
| RCIA | Velocity | 0.03(0.02,0.19) | 0.15(0.04,0.30) | 0.10(0.02,0.21) | 0.053 |
|  | Pressure | 7933.93±1428.34 | 7375.50±439.83 | -558.43(-1257.53,140.67) | 0.111 |
|  | WSS | 4.79(2.34,14.37) | 6.63(2.81,10.25) | 1.48(-2.55,5.80) | 0.904 |
|  | TAWSS | 4.95(2.48,16.06) | 6.17(2.65,11.14) | 1.10(-3.92,5.17) | 0.841 |
|  | OSI | 0.001(0,0.003) | 0(0,0.001) | -0.001(-0.002,0.00) | 0.015 |
|  | RRT | 0.20(0.06,0.41) | 0.16(0.09,0.38) | -0.02(-0.13,0.13) | 0.546 |

Group C: Hemodynamics at the last follow-up in the dilated group. Group F: Normal control group. TEVAR, thoracic endovascular aortic repair. MD, Median difference.95% CI, 95% confidence interval. BCT, brachiocephalic trunk; LCCA, left common carotid artery; LSA, left subclavian artery; SMA, superior mesenteric artery; LRA, left renal artery; RRA, right renal artery; IMA, inferior mesenteric artery; LCIA, left common iliac artery; RCIA, right common iliac artery. WSS, wall shear stress; TAWSS, time-averaged wall shear stress; OSI, oscillatory shear index; RRT, relative residence time. Velocity is presented in m/s, pressure in Pa, and WSS in Pa. Continuous data were expressed as mean ± standard deviation or median and interquartile range. Categorical variables were reported as absolute values and percentages.
