# Supplementary material for: Hemodynamic factors of aortic dilatation after thoracic endovascular aortic repair for type-B aortic dissection
Source: Front Bioeng Biotechnol. 2026 Apr 22;14:1780047. doi: 10.3389/fbioe.2026.1780047 (PMC13143993; doi:10.3389/fbioe.2026.1780047)
Supplement: Supplementary file 8 [file Table9.docx]

**Supplementary Table 9 1-week post-TEVAR hemodynamics in the nondilated group versus the control group**

| Location | Variable | Group E(n=19) | Group F(n=19) | MD (95% CI) | P value |
| --- | --- | --- | --- | --- | --- |
| BCT | Velocity | 0.03(0.01,0.07) | 0.09(0.05,0.13) | 0.05(0.002,0.08) | 0.044 |
|  | Pressure | 14164.99±12553.67 | 7599.91±356.60 | -6565.08(-12621.90,-508.26) | 0.035 |
|  | WSS | 1.28(0.39,2.10) | 4.17(1.15,5.31) | 2.41(1.11,3.85) | 0.005 |
|  | TAWSS | 1.48(0.28,2.58) | 4.25(1.55,4.65) | 1.98(0.63,3.16) | 0.004 |
|  | OSI | 0.01(0,0.04) | 0.002(0,0.04) | 0.00(-0.01,0.01) | 0.492 |
|  | RRT | 0.68(0.39,1.35) | 0.25(0.22,1.09) | -0.27(-0.97,-0.13) | 0.024 |
| LCCA | Velocity | 0.04(0.02,0.10) | 0.03(0.02,0.13) | -0.01(-0.03,0.02) | 0.687 |
|  | Pressure | 13988.33±12150.58 | 7569.82±356.22 | -6418.51(-12283.23,-553.78) | 0.034 |
|  | WSS | 1.16(0.51,2.56) | 0.99(0.81,3.40) | 0.46(-1.02,1.63) | 0.717 |
|  | TAWSS | 1.53(0.62,2.70) | 1.74(0.80,2.53) | 0.48(-0.75,1.20) | 0.601 |
|  | OSI | 0.01(0,0.04) | 0.01(0.002,0.04) | 0.01(-0.01,0.03) | 0.687 |
|  | RRT | 0.71(0.37,4.08) | 0.62(0.41,1.66) | -0.03(-0.85,0.82) | 0.573 |
| LSA | Velocity | 0.05(0.02,0.09) | 0.04(0.04,0.06) | 0.01(-0.04,0.03) | 0.872 |
|  | Pressure | 13908.68±12055.39 | 7550.29±358.58 | -6358.39(-12177.17,-539.61) | 0.034 |
|  | WSS | 1.20(0.48,2.12) | 1.54(0.59,2.81) | 0.38(-0.81,1.18) | 0.334 |
|  | TAWSS | 1.66(0.58,2.64) | 1.24(0.90,3.79) | 0.42(-0.07,1.66) | 0.212 |
|  | OSI | 0.02(0,0.03) | 0.04(0.01,0.17) | 0.02(0.001,0.13) | 0.035 |
|  | RRT | 0.63(0.39,2.11) | 0.92(0.29,1.83) | -0.20(-0.38,0.82) | 0.573 |
| Celiac trunk | Velocity | 0.24(0.07,0.33) | 0.03(0.03,0.11) | -0.14(-0.23,-0.06) | 0.001 |
|  | Pressure | 8920.65±1405.44 | 7510.09±360.37 | -1410.56(-2112.25,-708.86) | 0.001 |
|  | WSS | 4.82(1.34,8.83) | 1.08(0.43,2.46) | -3.37(-4.70,0.45) | 0.251 |
|  | TAWSS | 4.57(2.26,8.51) | 1.33(0.34,2.27) | -3.63(-6.53,-1.01) | 0.002 |
|  | OSI | 0.002(0,0.02) | 0.001(0,0.02) | 0.00(-0.01,0.001) | 0.875 |
|  | RRT | 0.23(0.12,0.45) | 0.76(0.44,3.05) | 0.32(0.12,1.32) | 0.012 |
| SMA | Velocity | 0.18(0.09,0.42) | 0.03(0.02,0.11) | -0.13(-0.21,-0.07) | 0.001 |
|  | Pressure | 9062.77±1390.25 | 7509.93±370.65 | -1552.85(-2243.92,-861.77) | 0.001 |
|  | WSS | 6.42(1.99,11.06) | 1.27(0.44,2.95) | -4.21(-8.13,-2.10) | 0.001 |
|  | TAWSS | 8.11(1.20,10.50) | 1.12(0.50,2.57) | -5.44(-9.29,-3.26) | 0.002 |
|  | OSI | 0.001(0,0.003) | 0.003(0.001,0.02) | 0.001(-0.002,0.01) | 0.155 |
|  | RRT | 0.12(0.10,0.84) | 0.93(0.39,1.99) | 0.61(0.21,1.19) | 0.147 |
| LRA | Velocity | 0.066(0.028,0.152) | 0.04(0.03,0.07) | 0.00(-0.10,0.03) | 0.472 |
|  | Pressure | 9055.07±1334.25 | 7498.38±382.51 | -1556.69(-2234.34,-879.05) | 0.001 |
|  | WSS | 2.17(0.43,6.82) | 2.04(1.05,3.80) | -0.37(-3.06,1.70) | 0.398 |
|  | TAWSS | 2.30(0.37,6.79) | 3.01(1.16,3.96) | -0.24(-2.85,1.33) | 0.421 |
|  | OSI | 0.02(0.001,0.05) | 0.001(0.001,0.01) | -0.02(-0.03,0.001) | 0.071 |
|  | RRT | 0.46(0.15,2.69) | 0.48(0.27,0.88) | 0.003(-0.48,0.15) | 0.457 |
| RRA | Velocity | 0.09(0.02,0.12) | 0.04(0.02,0.06) | -0.04(-0.08,0.01) | 0.117 |
|  | Pressure | 8549.70±2429.54 | 7501.04±371.53 | -1048.66(-2248.58,151.27) | 0.083 |
|  | WSS | 3.64(1.25,10.99) | 2.37(1.57,4.20) | -0.91(-6.80,1.47) | 0.117 |
|  | TAWSS | 4.37(0.80,7.96) | 2.67(1.61,4.51) | -1.08(-5.74,0.69) | 0.136 |
|  | OSI | 0.005(0.002,0.02) | 0(0,0.003) | -0.004(-0.01,-0.001) | 0.005 |
|  | RRT | 0.23(0.13,1.25) | 0.38(0.22,0.62) | 0.13(-0.14,0.36) | 0.778 |
| IMA | Velocity | 0.05(0.02,0.16) | 0.03(0.02,0.04) | -0.02(-.011,0.01) | 0.070 |
|  | Pressure | 8435.11±1133.46 | 7424.77±430.94 | -1010.34(-1579.55,-441.13) | 0.002 |
|  | WSS | 3.55(1.18,15.34) | 1.24(0.77,2.70) | -2.31(-6.95,0.47) | 0.011 |
|  | TAWSS | 3.40(0.67,6.92) | 1.72(0.77,2.70) | -1.16(-5.88,0.66) | 0.084 |
|  | OSI | 0.003(0,0.01) | 0.002(0,0.011) | -0.001(-0.003,0.003) | 0.794 |
|  | RRT | 0.30(0.150,1.54) | 0.59(0.37,1.31) | 0.23(-0.42,0.53) | 0.872 |
| LCIA | Velocity | 0.12(0.05,0.37) | 0.16(0.06,0.31) | 0.02(-0.11,0.12) | 0.809 |
|  | Pressure | 8229.17±897.48 | 7344.30±488.06 | -884.88(-1358.65,-411.11) | 0.001 |
|  | WSS | 9.68(2.19,21.69) | 7.53(3.97,11.24) | -3.27(-8.43,1.57) | 0.136 |
|  | TAWSS | 10.00(2.14,18.60) | 7.67(4.21,11.40) | -3.83(-7.06,2.87) | 0.227 |
|  | OSI | 0.001(0,0.005) | 0(0,0.001) | 0.00(-0.01,0.00) | 0.221 |
|  | RRT | 0.10(0.05,0.47) | 0.13(0.09,0.24) | 0.03(-0.10,0.11) | 0.968 |
| RCIA | Velocity | 0.13(0.07,0.22) | 0.15(0.04,0.30) | -0.01(-0.09,0.12) | 0.658 |
|  | Pressure | 8170.14±843.71 | 7375.50±439.83 | -794.64(-1213.03,-376.26) | 0.001 |
|  | WSS | 7.63(3.39,13.84) | 6.63(2.81,10.25) | -1.55(-5.07,4.98) | 0.658 |
|  | TAWSS | 6.97(2.03,12.68) | 6.17(2.65,11.14) | 0.69(-5.45,3.81) | 0.968 |
|  | OSI | 0.001(0,0.006) | 0(0,0.001) | 0.00(-0.002,0.00) | 0.023 |
|  | RRT | 0.14(0.08,0.50) | 0.16(0.09,0.38) | -0.03(-0.09,0.09) | 0.587 |

Group E: Hemodynamics at 1-week post-TEVAR in the nondilated group. Group F: Normal control group. TEVAR, thoracic endovascular aortic repair. MD, Median difference.95% CI, 95% confidence interval. BCT, brachiocephalic trunk; LCCA, left common carotid artery; LSA, left subclavian artery; SMA, superior mesenteric artery; LRA, left renal artery; RRA, right renal artery; IMA, inferior mesenteric artery; LCIA, left common iliac artery; RCIA, right common iliac artery. WSS, wall shear stress; TAWSS, time-averaged wall shear stress; OSI, oscillatory shear index; RRT, relative residence time. Velocity is presented in m/s, pressure in Pa, and WSS in Pa. Continuous data were expressed as mean ± standard deviation or median and interquartile range. Categorical variables were reported as absolute values and percentages.
