# Supplementary material for: Hemodynamic factors of aortic dilatation after thoracic endovascular aortic repair for type-B aortic dissection
Source: Front Bioeng Biotechnol. 2026 Apr 22;14:1780047. doi: 10.3389/fbioe.2026.1780047 (PMC13143993; doi:10.3389/fbioe.2026.1780047)
Supplement: Supplementary file 9 [file Table2.docx]

**Supplementary Table 2 Baseline characteristics and inlet blood flow velocity**

| Variable | Normal control group(n=19) | pre-TEVAR group(n=10) | post-TEVAR group(n=12) | P value | F/𝜒2 |
| --- | --- | --- | --- | --- | --- |
| Male | 10(52.63) | 9(90.00) | 10(83.33) | 0.057 | 5.721 |
| Age(year) | 62.37±6.78 | 61.42±10.74 | 64.2±11.33 | 0.780 | 0.251 |
| Maximum blood flow velocity(cm/s) | 84.02±17.95 | 69.58±10.78 | 77.66±20.26 | 0.113 | 2.312 |
| Minimum blood flow velocity(cm/s) | 12.51±3.52 | 13.71±2.64 | 13.37±2.55 | 0.556 | 0.597 |
| Average blood flow velocity(cm/s) | 48.26±9.91 | 41.64±6.36 | 45.52±10.93 | 0.217 | 1.592 |

TEVAR, Thoracic endovascular aortic repair. Continuous data were expressed as mean ± standard deviation. Categorical variables were expressed as absolute values and percentages.
