# Supplementary material for: Hemodynamic factors of aortic dilatation after thoracic endovascular aortic repair for type-B aortic dissection
Source: Front Bioeng Biotechnol. 2026 Apr 22;14:1780047. doi: 10.3389/fbioe.2026.1780047 (PMC13143993; doi:10.3389/fbioe.2026.1780047)
Supplement: Supplementary file 10 [file Table5.docx]

**Supplementary Table 5 Pre-TEVAR hemodynamics in the dilated group versus the control group**

| Location | Variable | Group A(n=19) | Group F(n=19) | MD (95% CI) | P value |
| --- | --- | --- | --- | --- | --- |
| BCT | Velocity | 0.04(0.02,0.14) | 0.09(0.05,0.13) | -0.00(-0.04,0.07) | 0.936 |
|  | Pressure | 9367.65(8014.62,9615.39) | 7599.91±356.60 | -4002.73(-9700.78,1695.31) | ＜0.001 |
|  | WSS | 1.06(0.73,2.85) | 4.17(1.15,5.31) | 1.67(-0.02,3.63) | 0.243 |
|  | TAWSS | 1.79(0.87,3.12) | 4.25(1.55,4.65) | 0.57(-0.04,2.42) | 0.198 |
|  | OSI | 0.002(0,0.018) | 0.002(0,0.04) | 0.00(0.00,0.02) | 0.601 |
|  | RRT | 0.56(0.32,1.16) | 0.25(0.22,1.09) | -0.22(-0.54,-0.01) | 0.036 |
| LCCA | Velocity | 0.02(0.01,0.16) | 0.03(0.02,0.13) | 0.002(-0.01,0.04) .,lop | 0.809 |
|  | Pressure | 9254.43(8054.42,9545.90) | 7569.82±356.22 | -1642.56(-2059.44,-570.64) | ＜0.001 |
|  | WSS | 1.18(0.59,7.17) | 0.99(0.81,3.40) | -0.18(-2.96,0.64) | 0.314 |
|  | TAWSS | 1.64(0.59,5.94) | 1.74(0.80,2.53) | -0.70(-2.78,0.75) | 0.295 |
|  | OSI | 0.002(0,0.026) | 0.01(0.002,0.04) | 0.00(-0.02,0.02) | 0.376 |
|  | RRT | 0.61(0.17,1.85) | 0.62(0.41,1.66) | 0.09(-1.23,0.75) | 0.778 |
| LSA | Velocity | 0.05(0.03,0.14) | 0.04(0.04,0.06) | 0.00(-0.08,0.03) | 0.477 |
|  | Pressure | 10568.88±7782.08 | 7550.29±358.58 | -3018.59(-6787.91,750.72) | 0.110 |
|  | WSS | 2.63(1.08,8.20) | 1.54(0.59,2.81) | -0.67(-3.49,0.45) | 0.184 |
|  | TAWSS | 2.17(1.01,9.31) | 1.24(0.90,3.79) | 0.11(-4.51,0.50) | 0.314 |
|  | OSI | 0.002(0.001,0.054) | 0.04(0.01,0.17) | 0.02(-0.01,0.07) | 0.127 |
|  | RRT | 0.48(0.11,1.33) | 0.92(0.29,1.83) | 0.20(-0.64,0.83) | 0.777 |
| Celiac trunk | Velocity | 0.12(0.04,0.18) | 0.03(0.03,0.11) | -0.06(-1.10,-0.01) | 0.008 |
|  | Pressure | 8264.78±932.65 | 7510.09±360.37 | -754.69(-1262.61,-246377) | 0.006 |
|  | WSS | 4.21(2.53,9.47) | 1.08(0.43,2.46) | -2.09(-4.88,-1.45) | 0.003 |
|  | TAWSS | 6.81±6.19 | 1.33(0.34,2.27) | -3.25(-7.84,-1.98) | 0.001 |
|  | OSI | 0.001(0,0.02) | 0.001(0,0.02) | 0.00(0.00,0.00) | 0.918 |
|  | RRT | 0.27(0.11,0.37) | 0.76(0.44,3.05) | 0.70(0.26,1.22) | 0.033 |
| SMA | Velocity | 0.07(0.05,0.16) | 0.03(0.02,0.11) | -0.04(-0.13,0.01) | 0.150 |
|  | Pressure | 8140.82±734.05 | 7509.93±370.65 | -630.89(-1041.78,-220.01) | 0.005 |
|  | WSS | 5.71±5.70 | 1.27(0.44,2.95) | -2.26(-7.44,-0.15) | 0.020 |
|  | TAWSS | 5.72±5.58 | 1.12(0.50,2.57) | -2.45(-6.88,0.26) | 0.140 |
|  | OSI | 0.003(0,0.03) | 0.003(0.001,0.02) | 0.00(0.00,0.00) | 0.397 |
|  | RRT | 0.24(0.10,0.85) | 0.93(0.39,1.99) | 0.41(0.16,0.85) | 0.136 |
| LRA | Velocity | 0.12(0.03,0.22) | 0.04(0.03,0.07) | -0.07(-0.13,0.01) | 0.074 |
|  | Pressure | 8167.92±820.09 | 7498.38±382.51 | -663.94(-1143.68,-184.20) | 0.010 |
|  | WSS | 6.04±4.24 | 2.04(1.05,3.80) | -2.11(-6.50,0.70) | 0.059 |
|  | TAWSS | 5.65±3.98 | 3.01(1.16,3.96) | -2.79(-6.18,0.21) | 0.067 |
|  | OSI | 0.01(0,0.02) | 0.001(0.001,0.01) | 0.00(0.00,0.01) | 0.687 |
|  | RRT | 0.22(0.13,0.32) | 0.48(0.27,0.88) | 0.16(0.00,0.59) | 0.097 |
| RRA | Velocity | 0.04(0.02,0.08) | 0.04(0.02,0.06) | 0.00(-0.02,0.04) | 0.569 |
|  | Pressure | 8226.76±838.47 | 7501.04±371.53 | -722.02(-1216.11,-227.94) | 0.007 |
|  | WSS | 2.98(1.68,7.54) | 2.37(1.57,4.20) | -1.22(-2.37,0.77) | 0.231 |
|  | TAWSS | 5.70±5.24 | 2.67(1.61,4.51) | -0.92(-3.48,0.71) | 0.170 |
|  | OSI | 0.002(0.001,0.02) | 0(0,0.003) | 0.00(-0.01,0.00) | 0.157 |
|  | RRT | 0.30(0.12,0.71) | 0.38(0.22,0.62) | 0.07(-0.33,0.37) | 0.777 |
| IMA | Velocity | 0.05(0.02,0.12) | 0.03(0.02,0.04) | 0.00(-0.06,0.02) | 0.196 |
|  | Pressure | 8006.92±804.64 | 7424.77±430.94 | -574.09(-1068.11,-80.07) | 0.025 |
|  | WSS | 2.93(1.09,9.74) | 1.24(0.77,2.70) | 0.55(-4.18,1.23) | 0.136 |
|  | TAWSS | 3.24(1.60,8.74) | 1.72(0.77,2.70) | 0.44(-4.24,1.24) | 0.113 |
|  | OSI | 0.001(0,0.018) | 0.002(0,0.011) | 0.00(0.00,0.01) | 0.587 |
|  | RRT | 0.45±0.38 | 0.59(0.37,1.31) | 0.40(-0.19,1.13) | 0.136 |
| LCIA | Velocity | 0.06(0.04,0.18) | 0.16(0.06,0.31) | 0.04(0.00,0.25) | 0.090 |
|  | Pressure | 7825.59±894.20 | 7344.30±488.06 | -467.95(-1019.64,83.74) | 0.091 |
|  | WSS | 7.67(2.16,17.98) | 7.53(3.97,11.24) | 1.46(-2.96,3.94) | 0.948 |
|  | TAWSS | 7.91(0.99,18.84) | 7.67(4.21,11.40) | 2.60(-5.43,6.73) | 0.931 |
|  | OSI | 0.002(0,0.01) | 0(0,0.001) | 0.00(0.00,0.00) | 0.205 |
|  | RRT | 0.13(0.05,1.04) | 0.13(0.09,0.24) | -0.01(-0.35,0.09) | 0.360 |
| RCIA | Velocity | 0.04(0.03,0.10) | 0.15(0.04,0.30) | 0.10(0.01,0.24) | 0.019 |
|  | Pressure | 7818.44±813.63 | 7375.50±439.83 | -436.55(-933.66,60.55) | 0.081 |
|  | WSS | 4.86(3.44,9.44) | 6.63(2.81,10.25) | -0.78(-1.96,4.56) | 0.811 |
|  | TAWSS | 5.31(3.57,8.50) | 6.17(2.65,11.14) | -1.21(-2.09,7.22) | 0.845 |
|  | OSI | 0.001(0,0.01) | 0(0,0.001) | 0.00(0.00,0.00) | 0.066 |
|  | RRT | 0.20±0.12 | 0.16(0.09,0.38) | 0.04(-0.04,0.14) | 0.408 |

Group A: Pre-TEVAR hemodynamics in the dilated group. Group F: Normal control group. TEVAR, thoracic endovascular aortic repair. MD, Median difference.95% CI, 95% confidence interval. BCT, brachiocephalic trunk; LCCA, left common carotid artery; LSA, left subclavian artery; SMA, superior mesenteric artery; LRA, left renal artery; RRA, right renal artery; IMA, inferior mesenteric artery; LCIA, left common iliac artery; RCIA, right common iliac artery. WSS, wall shear stress; TAWSS, time-averaged wall shear stress; OSI, oscillatory shear index; RRT, relative residence time. Velocity is presented in m/s, pressure in Pa, and WSS in Pa. Continuous data were expressed as mean ± standard deviation or median and interquartile range. Categorical variables were reported as absolute values and percentages.
