# Supplementary material for: Hemodynamic factors of aortic dilatation after thoracic endovascular aortic repair for type-B aortic dissection
Source: Front Bioeng Biotechnol. 2026 Apr 22;14:1780047. doi: 10.3389/fbioe.2026.1780047 (PMC13143993; doi:10.3389/fbioe.2026.1780047)
Supplement: Supplementary file 11 [file Table3.docx]

**Supplementary Table 3 Hemodynamics in the dilated group: pre-TEVAR, 1-week post-TEVAR, and last follow-up**

| Location | Variable | Group A(n=19) | Group B(n=12) | Group C(n=19) | P value |
| --- | --- | --- | --- | --- | --- |
| BCT | Velocity | 0.04(0.02,0.14) | 0.04(0.02,0.10) | 0.05(0.04,0.09) | 0.920 |
|  | Pressure | 9367.65(8014.62,9615.39) | 8520.89±612.23 | 9513.99±3534.00 | 0.779 |
|  | WSS | 1.06(0.73,2.85) | 2.984(1.12,4.62) | 2.17(0.84,7.76) | 0.264 |
|  | TAWSS | 1.79(0.87,3.12) | 1.91(0.86,6.76) | 1.76(0.79,4.31) | 0.338 |
|  | OSI | 0.002(0,0.018) | 0.004(0,0.015) | 0.001(0,0.092) | 0.836 |
|  | RRT | 0.56(0.32,1.16) | 0.53(0.15,3.48) | 0.59(0.23,2.14) | 0.558 |
| LCCA | Velocity | 0.02(0.01,0.16) | 0.05(0.02,0.14) | 0.04(0.02,0.09) | 0.386 |
|  | Pressure | 9254.43(8054.42,9545.90) | 8557.80±695.58 | 9538.10±3122.78 | 0.338 |
|  | WSS | 1.18(0.59,7.17) | 2.63(0.917,5.12) | 1.23(0.69,5.27) | 0.174 |
|  | TAWSS | 1.64(0.59,5.94) | 1.78(1.07,6.06) | 1.64(0.63,3.35) | 0.472 |
|  | OSI | 0.002(0,0.026) | 0.002(0.001,0.025) | 0.002(0,0.057) | 0.828 |
|  | RRT | 0.61(0.17,1.85) | 0.57(0.17,0.99) | 0.61(0.30,1.59) | 0.717 |
| LSA | Velocity | 0.05(0.03,0.14) | 0.03(0.02,0.04) | 0.07(0.04,0.14) | 0.148 |
|  | Pressure | 10568.88±7782.08 | 8604.82±730.08 | 9892.98±4589.86 | 0.148 |
|  | WSS | 2.63(1.08,8.20) | 1.48(0.61,2.86) | 1.91(0.56,8.55) | 0.913 |
|  | TAWSS | 2.17(1.01,9.31) | 1.74(0.80,2.73) | 1.20(0.95,6.83) | 0.441 |
|  | OSI | 0.002(0.001,0.054) | 0.037(0,0.125) | 0.002(0,0.067) | 0.513 |
|  | RRT | 0.48(0.11,1.33) | 0.81(0.37,1.52) | 0.91(0.15,1.24) | 0.529 |
| Celiac trunk | Velocity | 0.12(0.04,0.18) | 0.06(0.02,0.17) | 0.13(0.04,0.25) | 0.338 |
|  | Pressure | 8264.78±932.65 | 7678.67±689.82 | 8113.18±973.74 | 0.013 |
|  | WSS | 4.21(2.53,9.47) | 5.95(1.89,8.59) | 7.72(2.00,14.60) | 0.264 |
|  | TAWSS | 6.81±6.19 | 6.93(1.53,8.68) | 6.48(3.01,14.67) | 0.920 |
|  | OSI | 0.001(0,0.015) | 0.002(0,0.030) | 0.002(0,0.017) | 0.622 |
|  | RRT | 0.27(0.11,0.37) | 0.15(0.12,0.86) | 0.19(0.07,0.33) | 0.920 |
| SMA | Velocity | 0.07(0.05,0.16) | 0.05(0.02,0.13) | 0.07(0.04,0.27) | 0.920 |
|  | Pressure | 8140.82±734.05 | 7743.82±749.03 | 8226.23±968.64 | 0.050 |
|  | WSS | 5.71±5.70 | 2.45(1.07,5.83) | 3.93(0.99,12.54) | 0.368 |
|  | TAWSS | 5.72±5.58 | 2.88(2.27,6.04) | 3.71(1.01,11.24) | 0.558 |
|  | OSI | 0.003(0,0.03) | 0.003(0.001,0.006) | 0(0,0.003) | 0.231 |
|  | RRT | 0.24(0.10,0.85) | 0.35(0.17,0.44) | 0.28(0.09,1.29) | 0.558 |
| LRA | Velocity | 0.12(0.03,0.22) | 0.06(0.04,0.15) | 0.06(0.02,0.18) | 0.695 |
|  | Pressure | 8167.92±820.09 | 7605.85±790.08 | 8482.31±1637.24 | 0.178 |
|  | WSS | 6.04±4.24 | 8.69(3.21,19.18) | 5.19(1.09,10.11) | 0.761 |
|  | TAWSS | 5.65±3.98 | 8.89(4.30,19.06) | 4.26(1.22,9.85) | 0.695 |
|  | OSI | 0.01(0,0.02) | 0.001(0,0.051) | 0.002(0,0.008) | 0.397 |
|  | RRT | 0.22(0.13,0.32) | 0.12(0.05,0.27) | 0.24(0.10,0.83) | 0.695 |
| RRA | Velocity | 0.036(0.019,0.083) | 0.043(0.028,0.131) | 0.035(0.021,0.108) | 0.320 |
|  | Pressure | 8226.76±838.47 | 7703.77±787.59 | 8350.60±1057.60 | 0.148 |
|  | WSS | 2.98(1.68,7.54) | 7.78(1.20,11.78) | 6.64(4.07,10.10) | 0.178 |
|  | TAWSS | 5.70±5.24 | 7.16(1.41,10.97) | 6.27(2.98,9.50) | 0.148 |
|  | OSI | 0.002(0.001,0.02) | 0.002(0,0.014) | 0.001(0,0.005) | 0.839 |
|  | RRT | 0.30(0.12,0.71) | 0.15(0.09,0.73) | 0.17(0.11,0.34) | 0.148 |
| IMA | Velocity | 0.05(0.02,0.12) | 0.06(0.04,0.20) | 0.02(0.01,0.05) | 0.417 |
|  | Pressure | 8006.92±804.64 | 7491.49±941.41 | 7736.95±621.29 | 0.513 |
|  | WSS | 2.93(1.09,9.74) | 6.18(2.84,9.93) | 3.31(1.40,15.81) | 0.856 |
|  | TAWSS | 3.24(1.60,8.74) | 5.32(2.91,10.52) | 2.27(1.87,8.27) | 0.753 |
|  | OSI | 0.001(0,0.018) | 0.002(0,0.007) | 0.001(0,0.010) | 0.522 |
|  | RRT | 0.45±0.38 | 0.19(0.10,0.37) | 0.44(0.12,0.57) | 0.661 |
| LCIA | Velocity | 0.06(0.04,0.18) | 0.03(0.02,0.10) | 0.05(0.02,0.14) | 0.534 |
|  | Pressure | 7825.59±894.20 | 7464.73±896.36 | 7888.64±1129.63 | 0.148 |
|  | WSS | 7.67(2.16,17.98) | 5.87(2.63,8.25) | 4.74(3.50,8.10) | 0.913 |
|  | TAWSS | 7.91(0.99,18.84) | 5.13(2.89,7.86) | 5.96(4.05,8.26) | 0.915 |
|  | OSI | 0.002(0,0.007) | 0.003(0,0.046) | 0.001(0,0.012) | 0.926 |
|  | RRT | 0.13(0.05,1.04) | 0.21(0.13,0.37) | 0.18(0.12,0.25) | 0.952 |
| RCIA | Velocity | 0.04(0.03,0.10) | 0.03(0.02,0.04) | 0.03(0.02,0.19) | 0.078 |
|  | Pressure | 7818.44±813.63 | 7307.73±747.89 | 7933.93±1428.34 | 0.234 |
|  | WSS | 4.86(3.44,9.44) | 7.15(2.27,11.72) | 4.79(2.34,14.37) | 0.761 |
|  | TAWSS | 5.31(3.57,8.50) | 6.32(2.15,10.33) | 4.95(2.48,16.06) | 0.913 |
|  | OSI | 0.001(0,0.005) | 0.001(0,0.018) | 0.001(0,0.003) | 0.261 |
|  | RRT | 0.20±0.12 | 0.19(0.10,0.52) | 0.20(0.06,0.41) | 0.933 |
| Distal tear | Velocity | 0.07(0.03,0.13) | 0.04(0.01,0.11) | 0.07(0.03,0.12) | 0.264 |
|  | Pressure | 7673.94±911.77 | 7255.10±874.82 | 7684.53±923.51 | 0.169 |
|  | WSS | 5.70±5.35 | 2.68(1.09,8.70) | 5.30(0.58,8.84) | 0.895 |
|  | TAWSS | 5.86±5.48 | 4.00(1.18,8.24) | 5.27(0.55,9.44) | 0.857 |
|  | OSI | 0.002(0,0.009) | 0.001(0.001,0.018) | 0.005(0,0.083) | 0.970 |
|  | RRT | 0.30(0.11,0.66) | 0.29(0.12,0.95) | 0.21(0.11,1.85) | 0.801 |

Group A: Pre‑TEVAR hemodynamics in the dilated group. Group B: Hemodynamics at 1-week post‑TEVAR in the dilated group. Group C: Hemodynamics at the last follow‑up in the dilated group. TEVAR, thoracic endovascular aortic repair. BCT, brachiocephalic trunk; LCCA, left common carotid artery; LSA, left subclavian artery; SMA, superior mesenteric artery; RRA, right renal artery; LRA, left renal artery; IMA, inferior mesenteric artery; RCIA, right common iliac artery; LCIA, left common iliac artery. WSS, wall shear stress; TAWSS, time‑averaged wall shear stress; OSI, oscillatory shear index; RRT, relative residence time. Velocity is presented in m/s, pressure in Pa, and WSS in Pa. Continuous data were expressed as mean ± standard deviation or median (interquartile range). Categorical variables were reported as absolute values and percentages.
