# Supplementary material for: Hemodynamic factors of aortic dilatation after thoracic endovascular aortic repair for type-B aortic dissection
Source: Front Bioeng Biotechnol. 2026 Apr 22;14:1780047. doi: 10.3389/fbioe.2026.1780047 (PMC13143993; doi:10.3389/fbioe.2026.1780047)
Supplement: Supplementary file 12 [file Table4.docx]

**Supplementary Table 4 Pre-TEVAR and 1-week post-TEVAR hemodynamics in the nondilated group**

| Location | Variable | Group D(n=19) | Group E (n=19) | MD (95% CI) | | P value | |
| --- | --- | --- | --- | --- | --- | --- | --- |
| BCT | Velocity | 0.02(0.01,0.05) | 0.03(0.01,0.07) | 0.01(-0.01,0.03) | | 0.433 | |
|  | Pressure | 8893.38±1487.33 | 14164.99±12553.67 | 5271.61(-1311.65,11854.87) | | 0.11 | |
|  | WSS | 1.11(0.48,1.63) | 1.28(0.39,2.10) | 0.19(-0.30,0.85) | | 0.376 | |
|  | TAWSS | 1.29(0.51,2.36) | 1.48(0.28,2.58) | -0.31(-1.28,0.62) | | 0.809 | |
|  | OSI | 0.01(0.001,0.13) | 0.01(0,0.04) | -0.003(-0.07,0.02) | | 0.344 | |
|  | RRT | 0.85(0.43,2.24) | 0.68(0.39,1.35) | 0.06(-0.90,0.32) | | 0.841 | |
| LCCA | Velocity | 0.03(0.02,0.05) | 0.04(0.02,0.10) | -0.001(-0.03,0.01) | | 0.727 | |
|  | Pressure | 8904.63±1450.95 | 13988.33±12150.58 | 5083.69(-1292.40,11460.79) | | 0.111 | |
|  | WSS | 0.92(0.29,1.90) | 1.16(0.51,2.56) | -0.02(-0.27,0.51) | | 0.573 | |
|  | TAWSS | 1.10(0.41,2.81) | 1.53(0.62,2.70) | -0.19(-0.42,0.78) | | 0.717 | |
|  | OSI | 0.01(0.004,0.03) | 0.01(0,0.04) | 0.000(-0.02,0.03) | | 0.925 | |
|  | RRT | 0.92(0.45,2.52) | 0.71(0.37,4.08) | 0.20(-0.46,2.09) | | 0.295 | |
| LSA | Velocity | 0.02(0.02,0.05) | 0.05(0.02,0.09) | 0.01(-0.01,0.03) | 0.376 | |  |
|  | Pressure | 8879.44±1418.32 | 13908.68±12055.39 | 5029.24(-1272.39,11330.87) | 0.111 | |  |
|  | WSS | 1.30(0.64,3.39) | 1.20(0.48,2.12) | -0.23(-0.64, -0.12) | 0.064 | |  |
|  | TAWSS | 1.64(0.84,3.09) | 1.66(0.58,2.64) | -0.48(-0.99,0.21) | 0.126 | |  |
|  | OSI | 0.01(0.001,0.10) | 0.02(0,0.03) | 0.00(-0.08,0.003) | 0.256 | |  |
|  | RRT | 0.73(0.37,1.34) | 0.63(0.39,2.11) | 0.16(-0.01,0.48) | 0.107 | |  |
| Celiac trunk | Velocity | 0.09(0.04,0.41) | 0.24(0.07,0.33) | -0.003(-0.07,0.13) | | 0.936 | |
|  | Pressure | 8463.65±653.91 | 8920.65±1405.44 | 456.99(-275.83,1189.84) | | 0.207 | |
|  | WSS | 2.37(1.15,14.86) | 4.82(1.34,8.83) | -0.64(-9.39,1.90) | | 0.260 | |
|  | TAWSS | 3.07(1.98,15.41) | 4.57(2.26,8.51) | -0.38(-8.29,1.34) | | 0.334 | |
|  | OSI | 0.001(0,0.01) | 0.002(0,0.02) | 0.00(-0.001,0.002) | | 0.517 | |
|  | RRT | 0.33(0.07,0.51) | 0.23(0.12,0.45) | 0.03(-0.26,0.09) | | 0.809 | |
| SMA | Velocity | 0.08(0.06,0.18) | 0.18(0.09,0.42) | 0.05(-0.03,0.15) | | 0.07 | |
|  | Pressure | 8498.41±785.94 | 9062.77±1390.25 | 564.37(-298.94,1427.67) | | 0.186 | |
|  | WSS | 2.78(1.49,8.29) | 6.42(1.99,11.06) | 2.66(-0.67,7.15) | | 0.126 | |
|  | TAWSS | 2.27(1.65,10.54) | 8.11(1.20,10.50) | 3.24(-1.58,7.74) | | 0.107 | |
|  | OSI | 0.01(0,0.04) | 0.001(0,0.003) | -0.004(-0.03,0.00) | | 0.036 | |
|  | RRT | 0.44(0.10,0.61) | 0.12(0.10,0.84) | -0.06(-0.37,0.04) | | 0.445 | |
| LRA | Velocity | 0.096(0.050,0.218) | 0.066(0.028,0.152) | -0.01(-0.07,0.04) | | 0.398 | |
|  | Pressure | 8645.17±659.40 | 9055.07±1334.25 | 409.91(-326.58,1146.40) | | 0.258 | |
|  | WSS | 2.57(1.38,6.17) | 2.17(0.43,6.82) | -0.02(-2.83,4.29) | | 0.936 | |
|  | TAWSS | 2.79(1.90,6.75) | 2.30(0.37,6.79) | -0.35(-2.98,3.50) | | 0.904 | |
|  | OSI | 0.01(0.001,0.04) | 0.02(0.001,0.05) | 0.00(-0.01,0.03) | | 0.407 | |
|  | RRT | 0.39(0.17,0.59) | 0.46(0.15,2.69) | 0.074(-0.20,1.31) | | 0.212 | |
| RRA | Velocity | 0.04(0.02,0.16) | 0.09(0.02,0.12) | -0.002(-0.02,0.07) | | 0.658 | |
|  | Pressure | 8633.09±698.17 | 8549.70±2429.54 | -83.38(-1198.61,1031.84) | | 0.877 | |
|  | WSS | 3.98(1.08,6.17) | 3.64(1.25,10.99) | 0.91(-0.33,5.36) | | 0.227 | |
|  | TAWSS | 3.97(1.85,6.51) | 4.37(0.80,7.96) | -0.01(-1.21,4.22) | | 0.601 | |
|  | OSI | 0.001(0,0.003) | 0.005(0.002,0.02) | 0.002(0.001,0.015) | | 0.111 | |
|  | RRT | 0.27(0.15,0.63) | 0.23(0.13,1.25) | -0.05(-0.10,0.30) | | 0.658 | |
| IMA | Velocity | 0.06(0.02,0.24) | 0.05(0.02,0.16) | -0.01(-0.09,0.05) | | 0.616 | |
|  | Pressure | 8301.16±584.83 | 8435.11±1133.46 | 84.52(-558.51,727.55) | | 0.785 | |
|  | WSS | 5.62(2.75,14.05) | 3.55(1.18,15.34) | -1.34(-8.89,1.51) | | 0.327 | |
|  | TAWSS | 6.89(2.54,9.46) | 3.40(0.67,6.92) | -2.07(-6.94, -0.46) | | 0.078 | |
|  | OSI | 0(0,0.02) | 0.003(0,0.01) | 0.00(-0.01,0.004) | | 0.625 | |
|  | RRT | 0.15(0.11,0.44) | 0.30(0.150,1.54) | 0.13(0.01,0.99) | | 0.058 | |
| LCIA | Velocity | 0.10(0.02,0.15) | 0.12(0.05,0.37) | 0.01(-0.004,0.11) | | 0.095 | |
|  | Pressure | 8306.75±784.46 | 8229.17±897.48 | -77.58(-590.03,434.87) | | 0.629 | |
|  | WSS | 9.39(2.34,15.97) | 9.68(2.19,21.69) | 2.06(-1.28,7.71) | | 0.212 | |
|  | TAWSS | 9.49(2.06,14.15) | 10.00(2.14,18.60) | 0.51(-2.35,11.88) | | 0.260 | |
|  | OSI | 0.001(0,0.003) | 0.001(0,0.005) | 0.00(-0.001,0.002) | | 0.401 | |
|  | RRT | 0.11(0.07,0.49) | 0.10(0.05,0.47) | -0.02(-0.09,0.02) | | 0.444 | |
| RCIA | Velocity | 0.06(0.02,0.13) | 0.13(0.07,0.22) | 0.05(-0.01,0.10) | | 0.136 | |
|  | Pressure | 7908.17±2031.96 | 8170.14±843.71 | 261.97(-854.51,1378.46) | | 0.628 | |
|  | WSS | 8.83(3.86,15.01) | 7.63(3.39,13.84) | 0.30(-8.81,4.92) | | 0.717 | |
|  | TAWSS | 8.49(3.68,13.31) | 6.97(2.03,12.68) | 0.05(-7.94,2.36) | | 0.748 | |
|  | OSI | 0.001(0,0.002) | 0.001(0,0.006) | 0.00(0.00,0.002) | | 0.441 | |
|  | RRT | 0.12(0.08,0.27) | 0.14(0.08,0.50) | -0.001(-0.13,0.11) | | 0.904 | |
| Distal tear | Velocity | 0.16(0.04,0.25) | 0.12(0.03,0.17) | 0.00(-0.04,0.04) | | 0.777 | |
|  | Pressure | 8116.79±639.31 | 7993.32±1109.31 | -123.47(-732.18,485.23) | | 0.675 | |
|  | WSS | 4.01(1.27,9.27) | 5.71(1.49,21.94) | 3.16(-0.13,6.61) | | 0.126 | |
|  | TAWSS | 4.68(1.30,8.39) | 5.83(1.77,21.48) | 0.88(-0.13,5.55) | | 0.159 | |
|  | OSI | 0.004(0,0.01) | 0.002(0,0.01) | 0.00(-0.01,0.003) | | 0.776 | |
|  | RRT | 0.22(0.12,0.79) | 0.19(0.05,0.60) | -0.06(-0.18,0.02) | | 0.573 | |

Group D: Pre-TEVAR hemodynamics in the nondilated group. Group E: Hemodynamics at 1-week post-TEVAR in the nondilated group. TEVAR, thoracic endovascular aortic repair. MD, Median difference.95% CI, 95% confidence interval. BCT, brachiocephalic trunk; LCCA, left common carotid artery; LSA, left subclavian artery; SMA, superior mesenteric artery; LRA, left renal artery; RRA, right renal artery; IMA, inferior mesenteric artery; LCIA, left common iliac artery; RCIA, right common iliac artery. WSS, wall shear stress; TAWSS, time-averaged wall shear stress; OSI, oscillatory shear index; RRT, relative residence time. Velocity is presented in m/s, pressure in Pa, and WSS in Pa. Continuous data were expressed as mean ± standard deviation or median and interquartile range. Categorical variables were reported as absolute values and percentages.
