# Supplementary material for: Hemodynamic factors of aortic dilatation after thoracic endovascular aortic repair for type-B aortic dissection
Source: Front Bioeng Biotechnol. 2026 Apr 22;14:1780047. doi: 10.3389/fbioe.2026.1780047 (PMC13143993; doi:10.3389/fbioe.2026.1780047)
Supplement: Supplementary file 13 [file Table11.docx]

**Supplementary Table 11 Hemodynamics in dilated and nondilated groups at 1-week post-TEVAR**

| Location | Variable | Group B(n=12) | Group E(n=19) | MD (95% CI) | P value |
| --- | --- | --- | --- | --- | --- |
| BCT | Velocity | 0.04(0.02,0.10) | 0.03(0.01,0.07) | 0.02(-0.02,0.04) | 0.146 |
|  | Pressure | 8520.89±612.23 | 14164.99±12553.67 | 7173.96(-2382.52,16730.45) | 0.127 |
|  | WSS | 2.984(1.12,4.62) | 1.28(0.39,2.10) | -2.06(-3.80,1.64) | 0.774 |
|  | TAWSS | 1.91(0.86,6.76) | 1.48(0.28,2.58) | -1.30(-4.39,1.19) | 0.272 |
|  | OSI | 0.004(0,0.015) | 0.01(0,0.04) | -0.001(-0.01,0.01) | 1.000 |
|  | RRT | 0.53(0.15,3.48) | 0.68(0.39,1.35) | 0.17(-2.06,1.97) | 0.814 |
| LCCA | Velocity | 0.05(0.02,0.14) | 0.04(0.02,0.10) | 0.003(-0.03,0.05) | 0.875 |
|  | Pressure | 8557.80±695.58 | 13988.33±12150.58 | 6861.96(-2468.78,16192.70) | 0.134 |
|  | WSS | 2.63(0.917,5.12) | 1.16(0.51,2.56) | -0.57(-5.03,1.56) | 0.388 |
|  | TAWSS | 1.78(1.07,6.06) | 1.53(0.62,2.70) | -0.40(-3.96,1.07 | 0.505 |
|  | OSI | 0.002(0.001,0.025) | 0.01(0,0.04) | 0.001(-0.001,0.01) | 0.754 |
|  | RRT | 0.57(0.17,0.99) | 0.71(0.37,4.08) | 0.10(-0.47,3.12) | 0.530 |
| LSA | Velocity | 0.03(0.02,0.04) | 0.05(0.02,0.09) | 0.004(-0.03,0.05) | 0.443 |
|  | Pressure | 8604.82±730.08 | 13908.68±12055.39 | 6902.68(-2372.51,16177.87) | 0.130 |
|  | WSS | 1.48(0.61,2.86) | 1.20(0.48,2.12) | -0.18(-1.10,1.10) | 0.814 |
|  | TAWSS | 1.74(0.80,2.73) | 1.66(0.58,2.64) | -0.39(-1.18,1.40) | 0.774 |
|  | OSI | 0.037(0,0.125) | 0.02(0,0.03) | 0.00(-0.07,0.02) | 1.000 |
|  | RRT | 0.81(0.37,1.52) | 0.63(0.39,2.11) | 0.05(-0.81,1.45) | 0.638 |
| Primary tear | Velocity | 0.10(0.03,0.19) | 0.17(0.08,0.34) | 0.06(-0.07,0.12) | 0.227 |
|  | Pressure | 7728.21±1196.74 | 9185.82±1878.48 | 1629.09(-299.77,3557.95) | 0.089 |
|  | WSS | 9.70(1.52,37.51) | 5.81(0.64,21.34) | -3.59(-18.79,5.80) | 0.549 |
|  | TAWSS | 10.03(1.23,33.65) | 5.21(0.65,19.79) | -10.44(-25.55,5.10) | 0.155 |
|  | OSI | 0.001(0,0.003) | 0.001(0,0.017) | 0.00(-0.001,0.02) | 0.310 |
|  | RRT | 0.15(0.03,0.93) | 0.20(0.05,1.73) | 0.02(-0.30,1.14) | 0.424 |
| Celiac trunk | Velocity | 0.06(0.02,0.17) | 0.24(0.07,0.33) | 0.08(0.03,0.18) | 0.039 |
|  | Pressure | 7678.67±689.82 | 8920.65±1405.44 | 1261.06(-32.23,2554.34) | 0.055 |
|  | WSS | 5.95(1.89,8.59) | 4.82(1.34,8.83) | 0.36(-5.17,1.67) | 0.583 |
|  | TAWSS | 6.93(1.53,8.68) | 4.57(2.26,8.51) | -0.01(-4.37,0.90) | 0.480 |
|  | OSI | 0.002(0,0.030) | 0.002(0,0.02) | 0.001(-0.02,0.02) | 0.646 |
|  | RRT | 0.15(0.12,0.86) | 0.23(0.12,0.45) | -0.001(-0.15,0.21) | 0.790 |
| SMA | Velocity | 0.05(0.02,0.13) | 0.18(0.09,0.42) | 0.09(0.001,0.37) | 0.034 |
|  | Pressure | 7743.82±749.03 | 9062.77±1390.25 | 1362.67(173.72,2551.61) | 0.028 |
|  | WSS | 2.45(1.07,5.83) | 6.42(1.99,11.06) | 2.08(-2.18,7.61) | 0.338 |
|  | TAWSS | 2.88(2.27,6.04) | 8.11(1.20,10.50) | 4.58(-3.33,7.03) | 0.388 |
|  | OSI | 0.003(0.001,0.006) | 0.001(0,0.003) | 0.00(-0.01,0.002) | 0.549 |
|  | RRT | 0.35(0.17,0.44) | 0.12(0.10,0.84) | -0.17(-0.29,0.89) | 0.302 |
| LRA | Velocity | 0.06(0.04,0.15) | 0.066(0.028,0.152) | -0.03(-0.10,0.11) | 0.427 |
|  | Pressure | 7605.85±790.08 | 9055.07±1334.25 | 1164.28(250.70,2077.85) | 0.016 |
|  | WSS | 8.69(3.21,19.18) | 2.17(0.43,6.82) | -1.34(-13.32,1.95) | 0.233 |
|  | TAWSS | 8.89(4.30,19.06) | 2.30(0.37,6.79) | -3.90(-12.51,4.17) | 0.607 |
|  | OSI | 0.001(0,0.051) | 0.02(0.001,0.05) | -0.001(-0.06,0.05) | 1.000 |
|  | RRT | 0.12(0.05,0.27) | 0.46(0.15,2.69) | 0.28(-0.36,1.27) | 1.000 |
| RRA | Velocity | 0.043(0.028,0.131) | 0.09(0.02,0.12) | -0.01(-0.14,0.05) | 0.820 |
|  | Pressure | 7703.767±787.593 | 8549.70±2429.54 | 1129.27(194.88,2063.67) | 0.021 |
|  | WSS | 7.775(1.197,11.782) | 3.64(1.25,10.99) | -1.34(-4.97,9.95) | 0.607 |
|  | TAWSS | 7.160(1.413,10.966) | 4.37(0.80,7.96) | -1.34(-5.32,6.78) | 0.691 |
|  | OSI | 0.002(0,0.014) | 0.005(0.002,0.02) | 0.001(-0.003,0.01) | 0.955 |
|  | RRT | 0.15(0.09,0.73) | 0.23(0.13,1.25) | 0.05(-0.56,0.88) | 0.650 |
| IMA | Velocity | 0.06(0.04,0.20) | 0.05(0.02,0.16) | -0.02(-0.10,0.03) | 0.369 |
|  | Pressure | 7491.49±941.41 | 8435.11±1133.46 | 844.65(-26.28,1715.57) | 0.056 |
|  | WSS | 6.18(2.84,9.93) | 3.55(1.18,15.34) | -1.49(-5.32,6.78) | 0.424 |
|  | TAWSS | 5.32(2.91,10.52) | 3.40(0.67,6.92) | -2.43(-6.32,1.34) | 0.180 |
|  | OSI | 0.002(0,0.007) | 0.003(0,0.01) | 0.00(-0.003,0.005) | 0.937 |
|  | RRT | 0.19(0.10,0.37) | 0.30(0.150,1.54) | 0.20(-0.18,1.26) | 0.221 |
| LCIA | Velocity | 0.03(0.02,0.10) | 0.12(0.05,0.37) | 0.05(-0.07,0.11) | 0.607 |
|  | Pressure | 7464.73±896.36 | 8229.17±897.48 | 530.49(-165.83,1226.81) | 0.125 |
|  | WSS | 5.87(2.63,8.25) | 9.68(2.19,21.69) | 3.74(-1.45,12.74) | 0.302 |
|  | TAWSS | 5.13(2.89,7.86) | 10.00(2.14,18.60) | 3.86(-1.97,13.85) | 0.369 |
|  | OSI | 0.003(0,0.046) | 0.001(0,0.005) | -0.004(-0.03,0.002) | 0.124 |
|  | RRT | 0.21(0.13,0.37) | 0.10(0.05,0.47) | -0.12(-0.35,-0.01) | 0.034 |
| RCIA | Velocity | 0.03(0.02,0.04) | 0.13(0.07,0.22) | 0.10(0.01,0.13) | 0.035 |
|  | Pressure | 7307.73±747.89 | 8170.14±843.71 | 654.60(0.74,1308.46) | 0.045 |
|  | WSS | 7.15(2.27,11.72) | 7.63(3.39,13.84) | -1.56(-4.47,6.90) | 0.608 |
|  | TAWSS | 6.32(2.15,10.33) | 6.97(2.03,12.68) | 0.32(-5.32,6.67) | 0.534 |
|  | OSI | 0.001(0,0.018) | 0.001(0,0.006) | 0.00(-0.12,0.00) | 0.549 |
|  | RRT | 0.19(0.10,0.52) | 0.14(0.08,0.50) | -0.06(-0.42,0.15) | 0.302 |
| Distal tear | Velocity | 0.04(0.01,0.11) | 0.12(0.03,0.17) | 0.09(-0.02,0.14) | 0.394 |
|  | Pressure | 7255.10±874.82 | 7993.32±1109.31 | 520.46(-224.39,1265.32) | 0.156 |
|  | WSS | 2.68(1.09,8.70) | 5.71(1.49,21.94) | -0.62(-5.09,6.80) | 0.691 |
|  | TAWSS | 4.00(1.18,8.24) | 5.83(1.77,21.48) | -0.09(-4.80,6.79) | 0.650 |
|  | OSI | 0.001(0.001,0.018) | 0.002(0,0.01) | 0.001(-0.03,0.04) | 0.955 |
|  | RRT | 0.29(0.12,0.95) | 0.19(0.05,0.60) | -0.05(-1.03,0.34) | 0.657 |

Group B: Hemodynamics at 1-week post‑TEVAR in the dilated group. Group E: Hemodynamics at 1-week post-TEVAR in the nondilated group. TEVAR, thoracic endovascular aortic repair. MD, Median difference.95% CI, 95% confidence interval. BCT, brachiocephalic trunk; LCCA, left common carotid artery; LSA, left subclavian artery; SMA, superior mesenteric artery; LRA, left renal artery; RRA, right renal artery; IMA, inferior mesenteric artery; LCIA, left common iliac artery; RCIA, right common iliac artery. WSS, wall shear stress; TAWSS, time-averaged wall shear stress; OSI, oscillatory shear index; RRT, relative residence time. Velocity is presented in m/s, pressure in Pa, and WSS in Pa. Continuous data were expressed as mean ± standard deviation or median and interquartile range. Categorical variables were reported as absolute values and percentages.
