# Supplementary material for: Hemodynamic factors of aortic dilatation after thoracic endovascular aortic repair for type-B aortic dissection
Source: Front Bioeng Biotechnol. 2026 Apr 22;14:1780047. doi: 10.3389/fbioe.2026.1780047 (PMC13143993; doi:10.3389/fbioe.2026.1780047)
Supplement: Supplementary file 14 [file Table6.docx]

**Supplementary Table 6 1-week post-TEVAR hemodynamics in the dilated group versus the control group**

| Location | Variable | Group B(n=12) | Group F(n=19) | MD (95% CI) | P value |
| --- | --- | --- | --- | --- | --- |
| BCT | Velocity | 0.04(0.02,0.10) | 0.09(0.05,0.13) | 0.06(-0.01,0.11) | 0.117 |
|  | Pressure | 8520.89±612.23 | 7599.91±356.60 | -932.08(-1439.46,-424.70) | 0.002 |
|  | WSS | 2.984(1.12,4.62) | 4.17(1.15,5.31) | -0.71(-3.20,3.88) | 0.875 |
|  | TAWSS | 1.91(0.86,6.76) | 4.25(1.55,4.65) | 0.12(-1.59,3.75) | 0.530 |
|  | OSI | 0.004(0,0.015) | 0.002(0,0.04) | -0.00(-0.01,0.02) | 0.683 |
|  | RRT | 0.53(0.15,3.48) | 0.25(0.22,1.09) | 0.03(-2.34,0.83) | 0.875 |
| LCCA | Velocity | 0.05(0.02,0.14) | 0.03(0.02,0.13) | -0.01(-0.07,0.06) | 0.937 |
|  | Pressure | 8557.80±695.58 | 7569.82±356.22 | -987.57(-1530.82,-444.32) | 0.002 |
|  | WSS | 2.63(0.917,5.12) | 0.99(0.81,3.40) | -1.45(-3.09,-0.08) | 0.028 |
|  | TAWSS | 1.78(1.07,6.06) | 1.74(0.80,2.53) | -0.92(-2.76,0.35) | 0.117 |
|  | OSI | 0.002(0.001,0.025) | 0.01(0.002,0.04) | 0.01(-0.002,0.05) | 0.158 |
|  | RRT | 0.57(0.17,0.99) | 0.62(0.41,1.66) | 0.18(-0.21,0.62) | 0.347 |
| LSA | Velocity | 0.03(0.02,0.04) | 0.04(0.04,0.06) | 0.007(-0.004,0.03) | 0.308 |
|  | Pressure | 8604.82±730.08 | 7550.29±358.58 | -10.9.41(-1597.23,-481.60) | 0.002 |
|  | WSS | 1.48(0.61,2.86) | 1.54(0.59,2.81) | -0.11(-1.65,1.41) | 0.937 |
|  | TAWSS | 1.74(0.80,2.73) | 1.24(0.90,3.79) | 0.22(-0.49,0.53) | 0.695 |
|  | OSI | 0.04(0,0.13) | 0.04(0.01,0.17) | 0.01(-0.063,0.03) | 0.295 |
|  | RRT | 0.81(0.37,1.52) | 0.92(0.29,1.83) | 0.02(-0.32,0.34) | 0.875 |
| Celiac trunk | Velocity | 0.06(0.02,0.17) | 0.03(0.03,0.11) | -0.004(-0.09,0.05) | 0.433 |
|  | Pressure | 7678.67±689.82 | 7510.09±360.37 | -144.59(-685.32,396.13) | 0.568 |
|  | WSS | 5.95(1.89,8.59) | 1.08(0.43,2.46) | -4.57(-6.70,-0.86) | 0.015 |
|  | TAWSS | 6.93(1.53,8.68) | 1.33(0.34,2.27) | -5.70(-6.79,-1.05) | 0.012 |
|  | OSI | 0.002(0,0.030) | 0.001(0,0.02) | 0.00(00.02,0.01) | 0.646 |
|  | RRT | 0.15(0.12,0.86) | 0.76(0.44,3.05) | 0.54(-0.17,2.94) | 0.071 |
| SMA | Velocity | 0.05(0.02,0.13) | 0.03(0.02,0.11) | -0.01(-0.07,0.06) | 0.564 |
|  | Pressure | 7743.82±749.03 | 7509.93±370.65 | -212.06(-783.77,359.65) | 0.432 |
|  | WSS | 2.45(1.07,5.83) | 1.27(0.44,2.95) | -2.03(-4.23,0.89) | 0.158 |
|  | TAWSS | 2.88(2.27,6.04) | 1.12(0.50,2.57) | -2.43(-4.79,0.28) | 0.060 |
|  | OSI | 0.003(0.001,0.006) | 0.003(0.001,0.02) | 0.00(-0.004,0.02) | 0.386 |
|  | RRT | 0.35(0.17,0.44) | 0.93(0.39,1.99) | 0.00(-0.004,0.02) | 0.386 |
| LRA | Velocity | 0.06(0.04,0.15) | 0.04(0.03,0.07) | -0.02(-0.09,0.02) | 0.117 |
|  | Pressure | 7605.85±790.08 | 7498.38±382.51 | -76.83(-666.15,512.50) | 0.780 |
|  | WSS | 8.69(3.21,19.18) | 2.04(1.05,3.80) | -6.47(-16.35,-2.16) | 0.006 |
|  | TAWSS | 8.89(4.30,19.06) | 3.01(1.16,3.96) | -4.85(-15.29,-2.93) | 0.004 |
|  | OSI | 0.001(0,0.051) | 0.001(0.001,0.01) | 0.00(-0.04,0.001) | 0.202 |
|  | RRT | 0.12(0.05,0.27) | 0.48(0.27,0.88) | 0.45(0.16,1.07) | 0.028 |
| RRA | Velocity | 0.043(0.028,0.131) | 0.04(0.02,0.06) | -0.002(-0.10,0.01) | 0.328 |
|  | Pressure | 7703.767±787.593 | 7501.04±371.53 | -168.54(-759.38,422.30) | 0.543 |
|  | WSS | 7.775(1.197,11.782) | 2.37(1.57,4.20) | -5.21(-9.72,1.15) | 0.248 |
|  | TAWSS | 7.160(1.413,10.966) | 2.67(1.61,4.51) | -4.86(-7.68,0.47) | 0.248 |
|  | OSI | 0.002(0,0.014) | 0(0,0.003) | -0.00(-0.01,0.00) | 0.097 |
|  | RRT | 0.15(0.09,0.73) | 0.38(0.22,0.62) | 0.34(-0.32,1.12) | 0.239 |
| IMA | Velocity | 0.06(0.04,0.20) | 0.03(0.02,0.04) | -0.02(-0.18,-0.00) | 0.009 |
|  | Pressure | 7491.49±941.41 | 7424.77±430.94 | -47.78(-754.75,659.19) | 0.883 |
|  | WSS | 6.18(2.84,9.93) | 1.24(0.77,2.70) | -2.25(-9.27,-1.27) | 0.004 |
|  | TAWSS | 5.32(2.91,10.52) | 1.72(0.77,2.70) | -2.81(-9.83,-1.72) | 0.008 |
|  | OSI | 0.002(0,0.007) | 0.002(0,0.011) | 0.00(-0.002,0.002) | 0.799 |
|  | RRT | 0.19(0.10,0.37) | 0.59(0.37,1.31) | 0.40(0.11,1.16) | 0.008 |
| LCIA | Velocity | 0.03(0.02,0.10) | 0.16(0.06,0.31) | 0.10(0.02,0.16) | 0.023 |
|  | Pressure | 7464.73±896.36 | 7344.30±488.06 | -66.93(-720.71,586.85) | 0.826 |
|  | WSS | 5.87(2.63,8.25) | 7.53(3.97,11.24) | 3.21(-0.06,6.94) | 0.083 |
|  | TAWSS | 5.13(2.89,7.86) | 7.67(4.21,11.40) | 3.14(-0.44,7.03) | 0.060 |
|  | OSI | 0.003(0,0.046) | 0(0,0.001) | -0.004(-0.03,0.00) | 0.053 |
|  | RRT | 0.21(0.13,0.37) | 0.13(0.09,0.24) | -0.09(-0.32,-0.002) | 0.071 |
| RCIA | Velocity | 0.03(0.02,0.04) | 0.15(0.04,0.30) | 0.06(0.01,0.18) | 0.012 |
|  | Pressure | 7307.73±747.89 | 7375.50±439.83 | 94.23(-450.86,639.31) | 0.711 |
|  | WSS | 7.15(2.27,11.72) | 6.63(2.81,10.25) | 0.86(-5.47,4.36) | 0.937 |
|  | TAWSS | 6.32(2.15,10.33) | 6.17(2.65,11.14) | 1.10(-3.50,4.81) | 0.638 |
|  | OSI | 0.001(0,0.018) | 0(0,0.001) | -0.001(-0.17,0.00) | 0.036 |
|  | RRT | 0.19(0.10,0.52) | 0.16(0.09,0.38) | -0.01(-0.25,0.09) | 0.638 |

Group B: 1-week post-TEVAR hemodynamics in the dilated group. Group F: Normal control group. TEVAR, thoracic endovascular aortic repair. MD, Median difference.95% CI, 95% confidence interval. BCT, brachiocephalic trunk; LCCA, left common carotid artery; LSA, left subclavian artery; SMA, superior mesenteric artery; LRA, left renal artery; RRA, right renal artery; IMA, inferior mesenteric artery; LCIA, left common iliac artery; RCIA, right common iliac artery. WSS, wall shear stress; TAWSS, time-averaged wall shear stress; OSI, oscillatory shear index; RRT, relative residence time. Velocity is presented in m/s, pressure in Pa, and WSS in Pa. Continuous data were expressed as mean ± standard deviation or median and interquartile range. Categorical variables were reported as absolute values and percentages.
