# Supplementary material for: Hemodynamic factors of aortic dilatation after thoracic endovascular aortic repair for type-B aortic dissection
Source: Front Bioeng Biotechnol. 2026 Apr 22;14:1780047. doi: 10.3389/fbioe.2026.1780047 (PMC13143993; doi:10.3389/fbioe.2026.1780047)
Supplement: Supplementary file 15 [file Table10.docx]

Supplementary Table 10 Pre-TEVAR hemodynamics in the dilated group versus the nondilated group

| Location | Variable | Group A(n=19) | Group D(n=19) | MD (95% CI) | P value |
| --- | --- | --- | --- | --- | --- |
| BCT | Velocity | 0.04(0.02,0.14) | 0.02(0.01,0.05) | -0.02(-0.11,-0.01) | 0.044 |
|  | Pressure | 9367.65(8014.62,9615.39) | 8893.38±1487.33 | -2709.26(-8437.47,3018.95) | 0.334 |
|  | WSS | 1.06(0.73,2.85) | 1.11(0.48,1.63) | -0.20(-1.63,0.62) | 0.212 |
|  | TAWSS | 1.79(0.87,3.12) | 1.29(0.51,2.36) | -0.51(-1.67,0.43) | 0.147 |
|  | OSI | 0.002(0,0.018) | 0.01(0.001,0.13) | 0.01(-0.001,0.08) | 0.157 |
|  | RRT | 0.56(0.32,1.16) | 0.85(0.43,2.24) | 0.33(-0.35,0.65) | 0.421 |
| LCCA | Velocity | 0.02(0.01,0.16) | 0.03(0.02,0.05) | -0.003(-0.07,0.01) | 0.469 |
|  | Pressure | 9254.43(8054.42,9545.90) | 8904.63±1450.95 | -3006.68(-9455.54,3442.18) | 0.340 |
|  | WSS | 1.18(0.59,7.17) | 0.92(0.29,1.90) | -0.69(-4.08,0.94) | 0.212 |
|  | TAWSS | 1.64(0.59,5.94) | 1.10(0.41,2.81) | 0.07(-1.94,1.17) | 0.314 |
|  | OSI | 0.002(0,0.026) | 0.01(0.004,0.03) | 0.001(-0.003,0.02) | 0.395 |
|  | RRT | 0.61(0.17,1.85) | 0.92(0.45,2.52) | 0.45(-0.45,1.99) | 0.235 |
| LSA | Velocity | 0.05(0.03,0.14) | 0.02(0.02,0.05) | -0.02(-0.05,0.01) | 0.359 |
|  | Pressure | 10568.88±7782.08 | 8879.44±1418.32 | -1689.44(-5499.83,2120.95) | 0.364 |
|  | WSS | 2.63(1.08,8.20) | 1.30(0.64,3.39) | -1.67(-3.81,1.47) | 0.648 |
|  | TAWSS | 2.17(1.01,9.31) | 1.64(0.84,3.09) | -1.64(-5.27,1.71) | 0.648 |
|  | OSI | 0.002(0.001,0.054) | 0.01(0.001,0.10) | 0.01(-0.001,0.08) | 0.167 |
|  | RRT | 0.48(0.11,1.33) | 0.73(0.37,1.34) | 0.33(-0.48,0.78) | 0.629 |
| Primary tear | Velocity | 0.08(0.03,0.25) | 0.10(0.05,0.26) | 0.001(-0.08,0.08) | 1.000 |
|  | Pressure | 11067.85±10535.19 | 8706.92±1650.15 | -2392.62(-7703.02,2917.78) | 0.355 |
|  | WSS | 4.78(1.24,14.01) | 4.97(1.44,8.32) | -0.75(-6.91,4.53) | 0.815 |
|  | TAWSS | 4.89(1.01,12.45) | 3.81(1.62,6.69) | -1.36(-6.04,3.17) | 0.446 |
|  | OSI | 0.001(0,0.013) | 0(0,0.01) | 0.00(-0.001,0.001) | 0.774 |
|  | RRT | 0.22(0.08,1.07) | 0.27(0.15,0.62) | 0.06(-0.59,0.43) | 0.879 |
| Celiac trunk | Velocity | 0.12(0.04,0.18) | 0.09(0.04,0.41) | 0.04(-0.10,0.27) | 0.469 |
|  | Pressure | 8264.78±932.65 | 8463.65±653.91 | 198.87(-276.99,674.73) | 0.392 |
|  | WSS | 4.21(2.53,9.47) | 2.37(1.15,14.86) | -0.31(-3.52,8.57) | 0.904 |
|  | TAWSS | 6.81±6.19 | 3.07(1.98,15.41) | -0.87(-5.57,6.68) | 0.748 |
|  | OSI | 0.001(0,0.015) | 0.001(0,0.01) | 0.00(-0.01,0.001) | 0.804 |
|  | RRT | 0.27(0.11,0.37) | 0.33(0.07,0.51) | 0.02(-0.21,0.36) | 0.520 |
| SMA | Velocity | 0.07(0.05,0.16) | 0.08(0.06,0.18) | 0.01(-0.06,0.11) | 0.747 |
|  | Pressure | 8140.82±734.05 | 8498.41±785.94 | 357.59(36.09,679.08) | 0.031 |
|  | WSS | 5.71±5.70 | 2.78(1.49,8.29) | 0.39(-1.60,2.12) | 1.000 |
|  | TAWSS | 5.72±5.58 | 2.27(1.65,10.54) | 1.20(-2.77,2.92) | 0.809 |
|  | OSI | 0.003(0,0.03) | 0.01(0,0.04) | 0.00(-0.003,0.003) | 0.977 |
|  | RRT | 0.24(0.10,0.85) | 0.44(0.10,0.61) | -0.03(-0.27,0.40) | 0.702 |
| LRA | Velocity | 0.12(0.03,0.22) | 0.096(0.050,0.218) | -0.01(0.09,0.07) | 0.811 |
|  | Pressure | 8167.92±820.09 | 8645.17±659.40 | 497.74(153.12,842.36) | 0.007 |
|  | WSS | 6.04±4.24 | 2.57(1.38,6.17) | -2.44(-4.43,1.96) | 0.238 |
|  | TAWSS | 5.65±3.98 | 2.79(1.90,6.75) | -2.29(-3.77,2.16) | 0.472 |
|  | OSI | 0.01(0,0.02) | 0.01(0.001,0.04) | 0.004(0.00,0.02) | 0.035 |
|  | RRT | 0.22(0.13,0.32) | 0.39(0.17,0.59) | 0.20(-0.11,0.37) | 0.133 |
| RRA | Velocity | 0.036(0.019,0.083) | 0.04(0.02,0.16) | 0.01(-0.004,0.05) | 0.096 |
|  | Pressure | 8226.764±838.469 | 8633.09±698.17 | 447.19(103.17,791.20) | 0.014 |
|  | WSS | 2.981(1.683,7.543) | 3.98(1.08,6.17) | 0.58(-3.83,3.32) | 0.815 |
|  | TAWSS | 5.703±5.238 | 3.97(1.85,6.51) | -0.47(-2.82,3.53) | 0.845 |
|  | OSI | 0.002(0.001,0.02) | 0.001(0,0.003) | -0.002(-0.01,0.001) | 0.302 |
|  | RRT | 0.30(0.12,0.71) | 0.27(0.15,0.63) | 0.08(-0.18,0.29) | 0.481 |
| IMA | Velocity | 0.05(0.02,0.12) | 0.06(0.02,0.24) | 0.02(-0.08,0.12) | 0.804 |
|  | Pressure | 8006.92±804.64 | 8301.16±584.83 | 325.37(-14.03,664.76) | 0.059 |
|  | WSS | 2.93(1.09,9.74) | 5.62(2.75,14.05) | 1.80(-0.84,8.04) | 0.454 |
|  | TAWSS | 3.24(1.60,8.74) | 6.89(2.54,9.46) | 0.32(-1.80,6.51) | 0.438 |
|  | OSI | 0.001(0,0.018) | 0(0,0.02) | 0.00(-0.002,0.02) | 0.549 |
|  | RRT | 0.45±0.38 | 0.15(0.11,0.44) | 0.00(-0.48,0.09) | 1.000 |
| LCIA | Velocity | 0.06(0.04,0.18) | 0.10(0.02,0.15) | -0.02(-0.07,0.06) | 0.481 |
|  | Pressure | 7825.59±894.20 | 8306.75±784.46 | 490.32(97.82,882.84) | 0.017 |
|  | WSS | 7.67(2.16,17.98) | 9.39(2.34,15.97) | 0.68(-4.66,5.84) | 0.845 |
|  | TAWSS | 7.91(0.99,18.84) | 9.49(2.06,14.15) | -0.65(-5.57,5.42) | 0.815 |
|  | OSI | 0.002(0,0.007) | 0.001(0,0.003) | -0.001(-0.003,0.00) | 0.367 |
|  | RRT | 0.13(0.05,1.04) | 0.11(0.07,0.49) | 0.01(-0.46,0.30) | 0.913 |
| RCIA | Velocity | 0.04(0.03,0.10) | 0.06(0.02,0.13) | -0.01(-0.02,0.05) | 0.629 |
|  | Pressure | 7818.44±813.63 | 7908.17±2031.96 | 85.40(-857.30,1028.11) | 0.851 |
|  | WSS | 4.86(3.44,9.44) | 8.83(3.86,15.01) | 2.06(-1.21,6.83) | 0.815 |
|  | TAWSS | 5.31(3.57,8.50) | 8.49(3.68,13.31) | 3.25(-1.70,6.29) | 0.481 |
|  | OSI | 0.001(0,0.005) | 0.001(0,0.002) | 0.00(-0.003,0.001) | 1.000 |
|  | RRT | 0.20±0.12 | 0.12(0.08,0.27) | -0.06(-0.13,0.08) | 0.586 |
| Distal tear | Velocity | 0.07(0.03,0.13) | 0.16(0.04,0.25) | -0.001(-0.05,0.17) | 1.000 |
|  | Pressure | 7673.94±911.77 | 8116.79±639.31 | 395.80(58.57,733.02) | 0.024 |
|  | WSS | 5.70±5.35 | 4.01(1.27,9.27) | 064(-1.42,3.55) | 0.629 |
|  | TAWSS | 5.86±5.48 | 4.68(1.30,8.39) | -0.17(-0.96,2.21) | 0.831 |
|  | OSI | 0.002(0,0.009) | 0.004(0,0.01) | 0.001(-0.003,0.003) | 0.804 |
|  | RRT | 0.30(0.11,0.66) | 0.22(0.12,0.79) | 0.01(-0.17,0.15) | 1.000 |

Group A: Pre‑TEVAR hemodynamics in the dilated group. Group D: Pre-TEVAR hemodynamics in the nondilated group. TEVAR, thoracic endovascular aortic repair. MD, Median difference.95% CI, 95% confidence interval. BCT, brachiocephalic trunk; LCCA, left common carotid artery; LSA, left subclavian artery; SMA, superior mesenteric artery; LRA, left renal artery; RRA, right renal artery; IMA, inferior mesenteric artery; LCIA, left common iliac artery; RCIA, right common iliac artery. WSS, wall shear stress; TAWSS, time-averaged wall shear stress; OSI, oscillatory shear index; RRT, relative residence time. Velocity is presented in m/s, pressure in Pa, and WSS in Pa. Continuous data were expressed as mean ± standard deviation or median and interquartile range. Categorical variables were reported as absolute values and percentages.
